# Beyond rational—biosensor-guided isolation of 100 independently evolved bacterial strain variants and comparative analysis of their genomes

**DOI:** 10.1186/s12915-023-01688-x

**Published:** 2023-09-04

**Authors:** Philipp T. Baumann, Michael Dal Molin, Hannah Aring, Karin Krumbach, Moritz-Fabian Müller, Bas Vroling, Philana V. van Summeren-Wesenhagen, Stephan Noack, Jan Marienhagen

**Affiliations:** 1 Institute of Bio- and Geosciences, Forschungszentrum Jülich, IBG-1: Biotechnology, 52425 Jülich, Germany; 2 Department I of Internal Medicine, University of Cologne, 50937 Cologne, Germany; 3 Center for Molecular Medicine Cologne (CMMC), University of Cologne, 50931 Cologne, Germany; 4 Bioprodict GmbH, Nieuwe Marktstraat 54E, 6511AA Nijmegen, The Netherlands; 5 SenseUp GmbH, C/O Campus Forschungszentrum, Wilhelm-Johnen-Strasse, 52428 Jülich, Germany; 6 Institute of Biotechnology, RWTH Aachen University, Worringer Weg 3, 52074 Aachen, Germany

**Keywords:** Biosensor, Fluorescence-activated cell sorting, High-throughput screening, Genome sequencing, Comparative genome analysis, Single-nucleotide polymorphism, Metabolic engineering, l-histidine, *Corynebacterium glutamicum*

## Abstract

**Background:**

In contrast to modern rational metabolic engineering, classical strain development strongly relies on random mutagenesis and screening for the desired production phenotype. Nowadays, with the availability of biosensor-based FACS screening strategies, these random approaches are coming back into fashion. In this study, we employ this technology in combination with comparative genome analyses to identify novel mutations contributing to product formation in the genome of a *Corynebacterium glutamicum* l-histidine producer. Since all known genetic targets contributing to l-histidine production have been already rationally engineered in this strain, identification of novel beneficial mutations can be regarded as challenging, as they might not be intuitively linkable to l-histidine biosynthesis.

**Results:**

In order to identify 100 improved strain variants that had each arisen independently, we performed > 600 chemical mutagenesis experiments, > 200 biosensor-based FACS screenings, isolated > 50,000 variants with increased fluorescence, and characterized > 4500 variants with regard to biomass formation and l-histidine production. Based on comparative genome analyses of these 100 variants accumulating 10–80% more l-histidine, we discovered several beneficial mutations. Combination of selected genetic modifications allowed for the construction of a strain variant characterized by a doubled l-histidine titer (29 mM) and product yield (0.13 C-mol C-mol^−1^) in comparison to the starting variant.

**Conclusions:**

This study may serve as a blueprint for the identification of novel beneficial mutations in microbial producers in a more systematic manner. This way, also previously unexplored genes or genes with previously unknown contribution to the respective production phenotype can be identified. We believe that this technology has a great potential to push industrial production strains towards maximum performance.

**Supplementary Information:**

The online version contains supplementary material available at 10.1186/s12915-023-01688-x.

## Background

Our world is confronted with increasing environmental pressure and drastic consequences of climate change due to the unrestrained overconsumption of our planet’s resources. In this context, implementation of a circular bio-based economy for the sustainable production of a broad range of food, health, and industrial products from biomass and carbon-rich waste streams could be key to solving this problem. Microorganisms are of great importance for the envisioned bio-based economy as they serve as cell factories for the efficient synthesis of the desired valuable products from cheap, carbon-rich substrates [1–3].

However, despite availability of precise genetic tools, and comprehensive knowledge of the microbial metabolism and its genetic regulation gained over the last decades, the rational development of such cell factories is still very labor-intensive and time-consuming. In addition, even after extensive metabolic engineering, the microbial catalyst (and thus the overall production process) usually operates far from the theoretical optimum (typically specified as theoretical maximum (product) yield *Y*_P/S_ [g_product_ g_substrate_^−1^]) — often for unknown reasons [4–6]. Hence, classical whole cell random mutagenesis and screening approaches made a comeback to improve production performance through the identification of novel genomic targets for metabolic engineering [3]. In this context, metabolite-responsive transcription factors are employed to construct intracellular, genetically encoded biosensors, which translate intracellular metabolite concentrations into graded and machine-readable output signals such as fluorescence [7, 8]. In combination with fluorescence-activated cell sorting (FACS), such biosensors allow for the rapid screening of several million strain variants at the single-cell level, without the need for individual cultivation [9]. Noteworthy, important biosensor parameters such as ligand specificity or biosensor sensitivity can be individually adapted to the desired application [10–12]. In the last years, biosensors proved to be powerful tools for the improvement of microbial production strains (and enzymes) in multiple screening campaigns [11, 13, 14]. Interestingly, preceding biosensor-based screening studies mainly focused on the identification of beneficial mutations starting from a library of randomly mutated wild-type cells [11, 15]. Thus, mostly already known mutations, and only occasionally novel beneficial mutational hot spots contributing to an improved production phenotype, were found [11, 15–19]. However, identification of highly desired novel hot spots in already rationally engineered microbial genomes is particularly challenging, since (i) random mutagenesis typically introduces several hundred mutations per genome and (ii) a direct physiological connection to the production phenotype is not always given. In this context, starting from an industrially relevant strain, which has been already rationally engineered for the synthesis of the desired product, combined with comparative genome analysis of improved strain variants would ease the identification of novel mutagenic hot spots in the evolved microbial genomes.

Lately, the sustainable production of the proteinogenic amino acid l-histidine with multiple applications in the food and pharmaceutical industries came into focus as the protein hydrolysis is still a major source of this essential amino acid [20]. Engineering of microorganisms for l-histidine production is challenging since the biosynthetic pathway leading to l-histidine shares the same precursor molecule with the pathways of purines, pyrimidines, tryptophan, and nicotinamide dinucleotides [21]. Furthermore, the l-histidine pathway intermediate 5-aminoimidazole-4-carboxamide ribonucleotide (AICAR) is channeled into purine biosynthesis, and l-histidine biosynthesis is also intertwined with the C1 metabolism [6]. Due to this complexity, the small number of published strains rationally engineered for l-histidine synthesis typically operates far below the theoretical maximum product yield of 0.44 g l-histidine g glucose^−1^ (when µ_max_ is set to 0.1) [6, 11, 20, 22–27].

In this study,﻿ we perform a biosensor-based directed evolution campaign in combination with automated comparative genome sequence analyses to identify novel genomic hot spots contributing to improved l-histidine synthesis in already extensively engineered *Corynebacterium glutamicum* production strains. In this context, we use the biosensor pSenHis in combination with FACS to isolate 100 improved l-histidine producers from 100 independently mutagenized cultures of the *C. glutamicum* production strain. After genome sequencing, we perform a comparative and combinatorial sequence analysis of the obtained 100 genomes to identify novel genomic hotspots with a potential contribution to improved l-histidine production of the isolated *C. glutamicum* variants. Finally, we reintroduce and combine identified beneficial mutations in the l-histidine production strain and evaluate the production performance in bioreactors under lab-scale conditions.

We believe that biosensor-based high-throughput screening technologies in combination with comparative analyses of multiple genomes will help to push strain engineering beyond its current limitations, and speed-up the transition to a bio-based economy.

## Results

### *C. glutamicum* CgHis1—engineered for l-histidine production

Initially, a rationally engineered l-histidine producing *C. glutamicum* strain had to be identified, which is suitable for the biosensor-based FACS screening campaign. *C. glutamicum* CgHis1, developed for l-histidine production by the SenseUP GmbH (Jülich, Germany), accumulates 11 mM l-histidine in defined medium containing 2% d-glucose within 48 h (microtiter plate format). The strain is derived from *C. glutamicum* ATCC 13032 and is characterized by the substitution of four promoters in the genome, which control the expression of all ten genes involved in l-histidine synthesis, by strong and constitutive promoters (P_trc_-*hisE-hisG(S143F /ΔC)*, P_trc_-*hisDCB*, P_tuf_-*hisHAFI*, and P_H36_-*hisN*). Moreover, *hisG* is replaced by *hisG(S143F/ΔC)* encoding for a feedback-resistant ATP phosphoribosyltransferase variant as first, rate-limiting enzyme of the l-histidine pathway [27]. Since the activities of HisG and phosphoribosyl-ATP pyrophosphatase (HisE) are known to limit l-histidine overproduction, both encoding genes are also episomally overexpressed (pHisOP1) [26, 27]. Furthermore, the promoter of the fructose bisphosphatase (*fbp*) is replaced by the strong and constitutive P_tuf_-promoter. Hence, flux through the pentose phosphate pathway is increased, providing ribose 5-phosphate as precursor molecule for l-histidine biosynthesis. To increase d-glucose uptake, *C. glutamicum* CgHis1 is also characterized by an in-frame deletion of the gene for the transcriptional regulator IolR, known to repress the expression of the gene for the sugar permease IolT1 [28, 29].

**Fig. 1:**
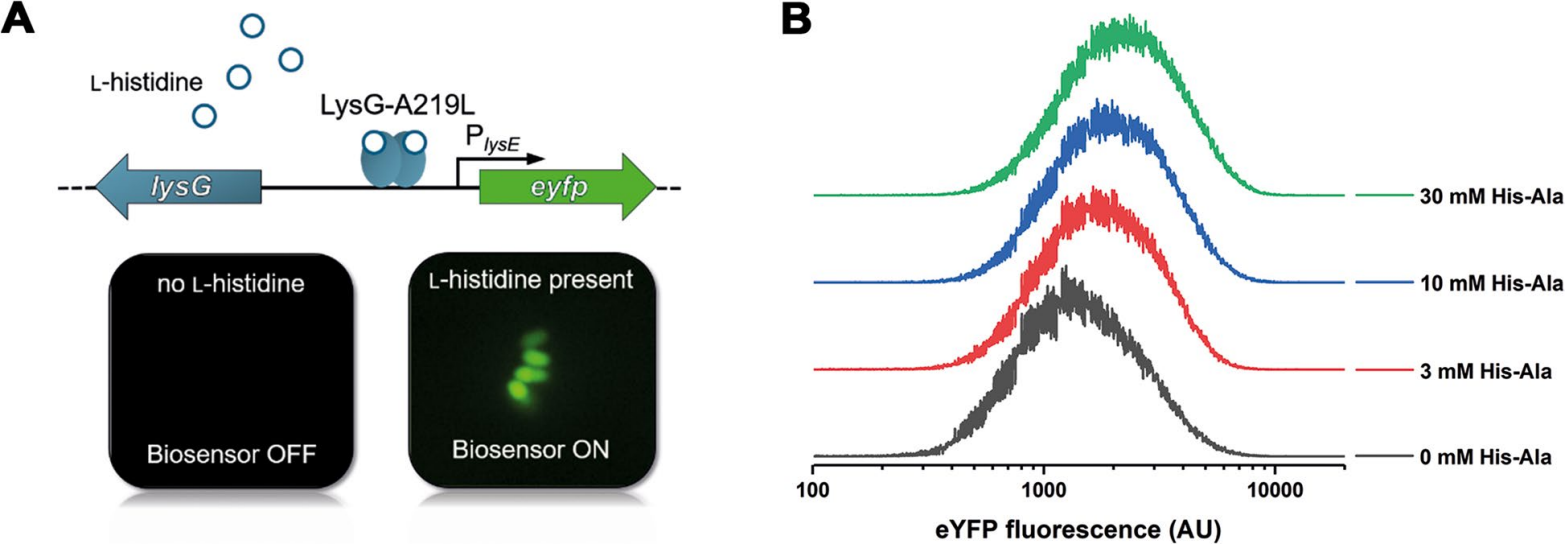
**A** Schematic representation of the plasmid-based pSenHis biosensor for intracellular detection of l-histidine in *C. glutamicum*. In the presence of elevated intracellular concentrations of l-histidine, the transcriptional activator LysG-A219L binds l-histidine and binds to its target promoter P_*lysE*_ to activate the expression of the *eyfp* gene encoding for the fluorescent reporter protein EYFP leading to fluorescent single cells. **B** Increased fluorescence response of single l-histidine-producing *C. glutamicum* CgHis2 cells during FACS upon supplementation of different His-Ala dipeptide concentrations**.** 0, 3, 10, and 30 mM l-His-l-Ala dipeptides were added to microtiter plate cultivations of *C. glutamicum* CgHis2 in defined CGXII medium with 2% d*-*glucose. In the middle of the exponential growth phase (after 6 h of cultivation), fluorescence intensities of 100,000 cells of each culture were analyzed by FACS. The depicted distributions represent standard deviations of the mean from three independent technical replicates showing the cell count at respective eYFP fluorescence (arbitrary units)

However, in order to use this strain in a FACS-based ultra-high-throughput screening campaign in combination with the plasmid-based l-histidine biosensor pSenHis, *C. glutamicum* CgHis1 had to be slightly modified. pSenHis relies on an engineered, l-lysine-insensitive variant of the LTTR-transcriptional regulator LysG from *C. glutamicum* ATCC 13032, which binds to its *lysE*-target promoter in response to the presence of intracellular l-histidine [11] (Fig. 1A). This activates the expression of the reporter gene *eyfp* coding for the enhanced yellow fluorescent protein leading to fluorescent single cells. Since the chromosome of *C. glutamicum* CgHis1 still harbors a wild-type version of *lysG*, *lysG* along with its target gene *lysE* were removed by in-frame deletion to avoid isolation of false-positive, l-lysine-accumulating *C. glutamicum* variants [15]. Furthermore, with the aim to reduce the number of plasmids, the pSenHis biosensor module was integrated into the plasmid pHisOP1 for episomal *hisEG* overexpression, yielding plasmid pSenHis[*hisEG*]. In microtiter plate cultivations, the generated strain *C. glutamicum* CgHis1 *ΔlysEG* pSenHis[*hisEG*] (further referred to as *C. glutamicum* CgHis2) grew comparable to *C. glutamicum* CgHis1 (pHisOP1) and accumulated the same amount of l-histidine (11.5 ± 0.2 mM, Additional file 1: Fig. S1). Subsequently, it was evaluated whether the biosensor pSenHis would be a suitable tool to distinguish mutated *C. glutamicum* CgHis2-variants with elevated intracellular product concentrations from the starting variant in the planned screening campaign. For this purpose, increased l-histidine synthesis capabilities were simulated in microtiter plate cultivations with online fluorescence measurements by supplementing l-His-l-Ala dipeptides known to be readily taken up and hydrolyzed by *C. glutamicum* [30, 31]. These experiments showed that the specific fluorescence of the cultures increased with increasing l-His-l-Ala dipeptide concentrations (Additional file 1: Fig. S2). This finding was also supported by FACS-analyses of single cells, as increasing l-His-l-Ala dipeptide concentrations shift the fluorescent population towards increased fluorescence intensity, indicating higher induction states of the biosensor (Fig. 1B). These experiments showed that pSenHis is suitable for the detection of *C. glutamicum* CgHis2 variants with improved l-histidine production capabilities compared to the starting variant.

Biosensor crosstalk between producing and non-producing cells due to product export (or diffusion) of the target compound by producing cells, and subsequent uptake by non-producing cells, might lead to the isolation of numerous false-positive strain variants and thus could negatively affect the performance of the biosensor-based screening campaign [14]. Although no l-histidine export or import system has been described for *C. glutamicum*, co-cultivation experiments with *C. glutamicum* CgHis2 and the *C. glutamicum* wild-type harboring pSenHis as reporter strain were conducted to monitor the fluorescence of both subpopulations via FACS (Additional file 1: Fig. S3). In none of the co-cultivations, *C. glutamicum* wild-type cells exhibited fluorescence, indicating biosensor crosstalk not to be relevant for this study.

### Multiplexed random mutagenesis and FACS screening

*C. glutamicum* CgHis2 has been already rationally engineered for l-histidine production. Thus, we expected to find beneficial mutations preferentially in genes unrelated to l-histidine biosynthesis during the biosensor-based FACS screening. However, since such new mutational hot spots contributing to l-histidine synthesis would be difficult to identify in a single, randomly mutated genome, we set out to isolate 100 l-histidine accumulating *C. glutamicum* CgHis2 variants from 100 independently mutagenized cultures by FACS. Potentially, beneficial mutations cluster in certain metabolic modules, pathways, or even single genes, which would ease their identification. Furthermore, independent mutagenesis, FACS screening, and characterization of 100 strain variants has the advantage to avoid isolation of genetically identical sister clones as it has been observed in previous studies [15].

Over the course of this biosensor-based FACS screening campaign, more than 600 *C. glutamicum* CgHis2 cultures were treated with different concentrations of the mutagen *N*-methyl-*N'*-nitro-*N*-nitrosoguanidine (MNNG) (Additional file 1: Fig. S4). Noteworthy, MNNG treatment resulted in high variance of the culture mortality rendering the process of generating appropriate cultures from which improved *C. glutamicum* CgHis2 variants could be reliably isolated challenging. In total, only 212 mutagenized *C. glutamicum* CgHis2 cultures showing a mortality rate < 99% were subsequently screened by FACS to isolate 100 independent strain variants with a significantly improved l-histidine titer. However, the cultivation time of the mutagenized *C. glutamicum* CgHis2 cultures and the time-point of FACS screening proved to be essential for minimizing the isolation of false-positive variants. False-positives were typically cells characterized by increased fluorescence without an increased production performance. This constitutive *eyfp*-expression independent from l-histidine accumulation could be traced back to mutations acquired in the biosensor module. These, however, could be sorted out during subsequent control experiments (individual cultivation and chromatographic analysis). Here, direct inoculation from glycerol stocks of mutagenized *C. glutamicum* CgHis2 cultures and FACS screening after 6 h of cultivation turned out to be most suitable for a reliable biosensor response. This was of great importance as the fluorescence response determines the FACS separation efficiency of improved l-histidine-producing cells from cells with the performance of the starting variant. Furthermore, with this short seedtrain ensuring a short overall cultivation time before FACS, “dilution” of the genetic diversity in the MNNG-mutagenized cultures due to cell division was minimized.

FACS-based enrichment strategies of strain variants with desired traits via repetitive cultivation and cell sorting had been shown to be useful in the past [11, 14, 32, 33]. Due to the necessity for high throughput to isolate 100 improved *C. glutamicum* CgHis2 variants, repetitive FACS enrichments were not deemed feasible for this study. Instead, single-cell sorting of the top 5% of fluorescent cells on agar plates was performed using FACS. Following this, 240 single cells were isolated from each of the 212 mutagenized *C. glutamicum* CgHis2 cultures — accounting for more than 50,000 strain variants in total.

**Fig. 2:**
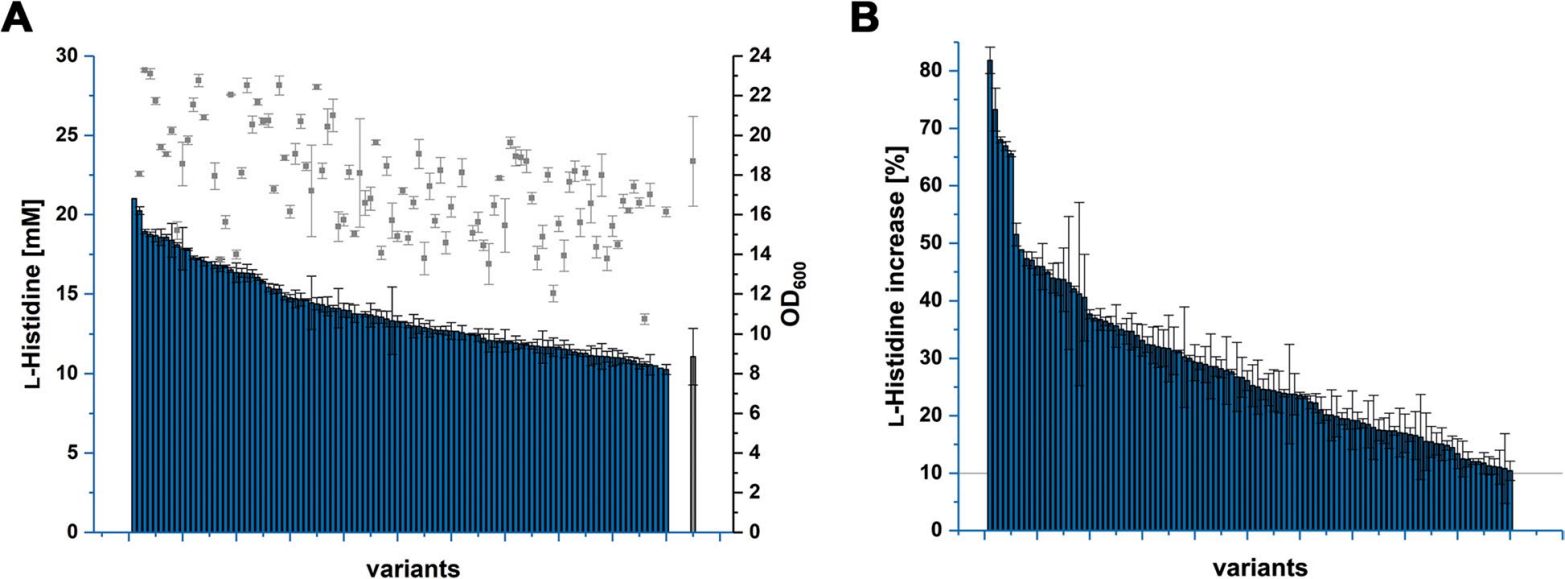
l-histidine accumulation and biomass formation of 100 FACS-isolated *C. glutamicum* CgHis2 variants. All variants were independently generated by chemical mutagenesis, independently isolated in a biosensor-based FACS screening and characterized in detail. **A** l-histidine titer and biomass after 48 h (second characterization step). Error bars of variants represent three independent technical replicates. *C. glutamicum* CgHis2 reference strain (grey) is displayed as mean over ten independent cultivation rounds. *Note:* Due to technical variation of humidity parameters during the cultivation, variants were evaluated only in comparison to the respective triplicate of controls on the same microtiter plate. Therefore, comparison of variants from all cultivation rounds was performed by **B** percentage improvement of l-histidine titers in comparison to the specific *C. glutamicum* CgHis2 starting strain control. An increased product formation of 10% was set as threshold. Data represent average values and standard deviation of three independent technical replicates

For the characterization of several thousand randomly picked clones, a two-step procedure was developed. Both steps comprised microtiter plate cultivations with end-point determination of the respective culture’s optical density and l-histidine concentration in the supernatant after 48 h of cultivation. In the first cultivation, promising *C. glutamicum* CgHis2 strain variants showing improved production performance were identified, whereas the second cultivation was performed in technical triplicates to characterize these preselected mutant strain variants in detail. Noteworthy, all steps included robotic sample preparation for accuracy, since only incremental improvements of the production performance were expected. A FACS-isolated *C. glutamicum* CgHis2 variant was considered to be improved, when the l-histidine titer increased by at least 10%. At the same time, a reduction of biomass formation by maximal 35% was defined as acceptable. This way, over 4600 and 364 variants were characterized in the first and second cultivation during the rescreening process, respectively. Thereby, the best 100 independently isolated l-histidine-overproducing *C. glutamicum* CgHis2 strain variants targeted could be identified (Fig. 2). Each of these 100 variants was the best-performing variant isolated from an independently mutagenized *C. glutamicum* CgHis2 culture. The overall best-performing isolate, *C. glutamicum* CgHis2 12–10-5–6, accumulated 82% more l-histidine in the supernatant compared to the *C. glutamicum* CgHis2 starting variant (20.9 ± 0.4 mM and 11.5 mM l-histidine; OD_600_ of 13.7 ± 0.1 and 18.4 ± 0.1, respectively).

### Genome sequencing and automated analysis of multiple genome sequences

**Fig. 3:**
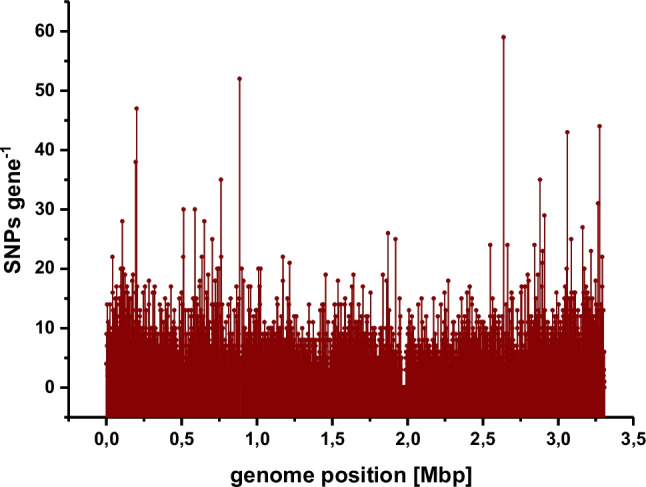
Total number of mutations per gene across 100 isolated *C. glutamicum* CgHis2 variants according to the position in the genome of *C. glutamicum*. High mutation rates of individual genes might indicate a link to the improved l-histidine production phenotype

Whole genome sequencing (WGS) delivered DNA sequences of all 100 independently evolved *C. glutamicum* CgHis2 strain variants with an increased production phenotype [34]**.** For identification of mutational hotspots with potential contribution to l-histidine production, the software tool “Fast Automated Analysis of Multiple Sequences” (FAAMS) was developed. The tool processes raw WGS data and maps them to the genome of the *C. glutamicum* ATCC 13032 wild-type strain. Quality control of the fastq files from the WGS is initially performed. Parameters such as total reads and read quality are collected and plotted for visual inspection. The fraction of passed reads is stated. A second quality control step indicates the fraction of reads allocated to the reference genome. FAAMS then identifies synonymous and nonsynonymous mutations in all genomes and attributes them to an open reading frame including an artificial 200 bp promoter region upstream if possible (terminator sequences were not taken into account). FAAMS also plots the total number of mutations per gene according to the respective gene’s position in the genome, since a high mutation rate could indicate a link to the increased l-histidine production phenotype (Fig. 3). In addition, FAAMS attributes mutations to central metabolic pathways with data obtained from KEGG (via the API). Hereby, genes corresponding to a pathway of interest could be highlighted (e.g., fatty acid synthesis). FAAMS provides mutations translated into amino acids on the protein level and attributes whether the mutations are synonymous, nonsynonymous, nonsense, small insertions and deletions, or mutations in the promoter region. For further information and analysis single clone data are available. One crucial aspect is the read coverage where large insertions (e.g., gene duplication) or deletions (e.g., gene deletions) are detected.

FAAMS identified 47–470 mutations per variant, evenly distributed across the individual genome (Additional file 1: Fig. S5) with an average mutation frequency of 180 mutations per genome. Probably due to the mutational spectrum of MNNG, which mostly leads to GC→AT transition mutations (Additional file 1: Fig. S6), and the short cultivation intervals between mutagenesis and FACS screening, no InDels could be found in any of the 100 genome sequences [35].

The mutation frequency in known engineering targets for l-histidine production such as l-histidine biosynthesis, purine biosynthesis, and C1 metabolism was found to be very low (Additional file 1: Table S3). Since mutations in *hisG* were sufficiently investigated elsewhere, identified mutations in this study were not investigated further [25, 27, 36, 37]. Therefore, novel beneficial mutations could now be revealed in mutational hot spots that go beyond common targets for engineering l-histidine production (Table 1).

**Table 1:** Most significant hotspot genes identified in the genomes of 100 independently generated *C. glutamicum* CgHis2 variants accumulating increased l-histidine concentrations

#	Locus tag	Gene	Enzyme	Nonsynonymous mutations in x variants
1	NCgl2409	*fasB*	Fatty acid synthase	24
2	NCgl2964		DEAD/DEAH box helicase	23
3	NCgl0802	*fasA*	Fatty acid synthase	22
4	NCgl2773	*pks*	Type 1 polyketide synthase	21
5	NCgl0184	*emb*	Arabinosyl transferase C	20
6	NCgl0181	*gltB*	Alpha subunit of glutamate synthase	19
7	NCgl0552		DNA segregation ATPase	19
8	NCgl2618	*cps*	Non-ribosomal peptide synthase	18
9	NCgl0040	*pknB*	Serine/threonine protein kinase	17
10	NCgl2959		Phosphoesterase	17
11	NCgl1737		Hypothetical membrane protein	17
12	NCgl2433	*dinG*	Probable ATP-dependent DNA helicase-related protein	15
13	NCgl2981		Hypothetical protein	15
14	NCgl0916	*ggtB*	Gamma-glutamyltranspeptidase	14
15	NCgl0705		Probable ATP-dependent helicase	13

### Identification of beneficial mutations and reverse engineering

Due to the high number of single-nucleotide polymorphisms (SNPs) in the genomes of the 100 isolated *C. glutamicum* CgHis2 variants and the low frequency of mutations in genes known to be involved in l-histidine biosynthesis, a comparative and combinatorial strategy had to be developed to identify beneficial mutations. Within the FAAMS dataset, all synonymous mutations and mutations exhibiting read frequencies below 50% were discarded first. Then all genes were ordered according to their number of nonsynonymous mutations. In the end, 71 genes were classified as “hotspot genes” potentially contributing to improved l-histidine production, when at least 10% of the isolated *C.* *glutamicum* CgHis2 strains carried mutations in this particular gene (Table 1, Additional file 1: Table S4, Additional file 1: Data S1).

No mutation occurred twice in any of the 100 strain variants, meaning that all mutations in the hotspot genes were located at different positions. Hence, three criteria were defined for selecting specific mutations with high probability of contribution to the increased l-histidine production phenotype. (I) *Hotspot abundance*: When selecting a SNP from a specific strain variant, the number of SNPs in other hotspot genes of this strain variant should be low. In such cases, the probability of the selected SNP to contribute to the improved l-histidine production phenotype was assumed to be higher. (II) *Growth performance*: The respective variant bearing the mutation in question should be characterized by a very high l-histidine accumulation in combination with no or only slightly reduced biomass formation. (III) *Metabolic consequences*: The (hypothetical) metabolic role of the respective protein bearing the mutation in question for the microbial metabolism, biomass formation and (if possible) l-histidine formation should be clear.

**Fig. 4:**
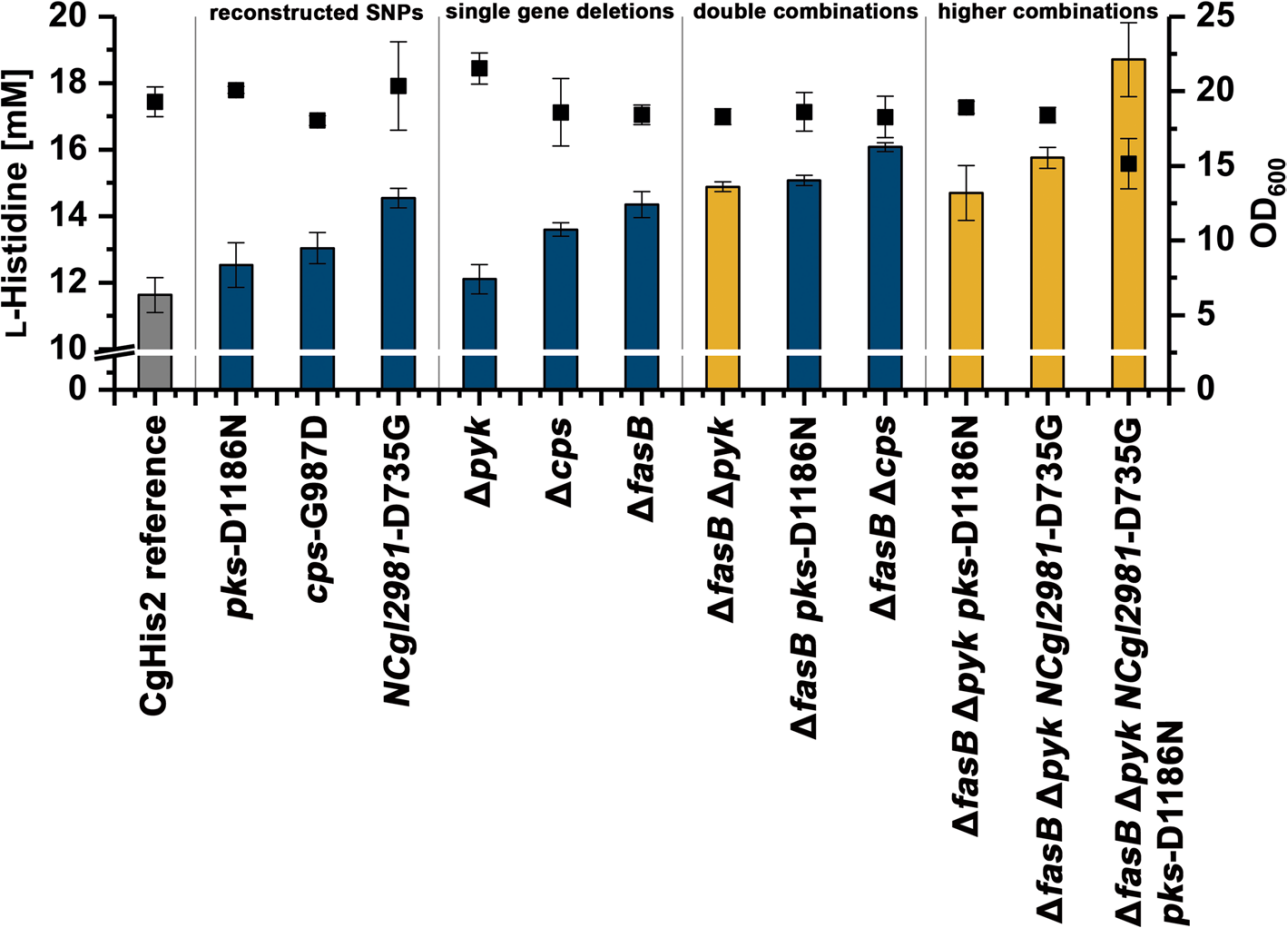
Growth and l-histidine production of *C. glutamicum* CgHis2 variants with different genomic modifications. *C. glutamicum* CgHis2 Δ*fasB* Δ*pyk* served as starting variant for the construction of various strains combining three or four genomic modifications, which exhibited significant increase in l-histidine production (yellow). Δ*pyk* always refers to the *pyk1* gene. *C. glutamicum* CgHis2 Δ*fasB* Δ*cps* served as starting variant for the construction of various strains with no improvement of product formation (Additional file 1: Fig. S9). Data represent average values and standard deviation of three independent technical replicates

Based on these selection criteria, 28 different point mutations in 20 hotspot genes were selected for individual introduction into the *C. glutamicum* CgHis2 starting strain and subsequent in-depth characterization with regard to growth and product formation (Additional file 1: Table S5). As a result of these experiments, three SNPs could be identified, which individually contribute to a significantly increased l-histidine production (Fig. 4). The first SNP was located in the *cps*-gene (*NCgl2618*), encoding a non-ribosomal peptide synthetase with so far unknown function in *C. glutamicum* (Additional file 1: Data S2)*.* When introduced into the genome of *C. glutamicum* CgHis2, this mutation leads to a G987D substitution in Cps and increased the l-histidine titer of this variant significantly by 12% (*p* = 0.009, Student’s *t* test). The polyketide synthase gene *pks* (*NCgl2773*) is involved in mycolic acid synthesis [38, 39]. When the SNP leading to a D1186N substitution in Pks was introduced into *C. glutamicum* CgHis2, the l-histidine titer increased by 8%. According to Student’s *t* test, this result is not significant (*p* = 0.06); however, two more cultivations showed significant contribution to l-histidine overproduction with increased titers of 6% (*p* = 0.01) and 9% (*p* = 0.02), respectively (Additional file 1: Data S2). The third nonsynonymous SNP with a significant impact on l-histidine production was located in the open reading frame of *NCgl2981*, a gene presumably encoding for a membrane glycoprotein of hitherto unknown function (Additional file 1: Data S2). Upon introduction of the mutation (leading to *NCgl2981*-D735G; Additional file 1: Fig. S7) into *C. glutamicum* CgHis2, the l-histidine titer of the constructed variant increased by 26%.

We hypothesized that random chemical mutagenesis predominantly results in reduced or even loss-of-function mutations and only few gain-of-function mutations [40]. Hence, individual in-frame deletions of *cps*, *pks*, and *NCgl2981* and 14 other genes in which promising SNPs were identified and were performed in *C. glutamicum* CgHis2 in addition to the individual reconstruction of the identified SNPs. We were able to generate 13 *C. glutamicum* CgHis2 variants with individual gene deletions from which *C. glutamicum* CgHis2 Δ*fasB*, *C.* *glutamicum* CgHis2 Δ*pyk1*, and *C. glutamicum* CgHis2 Δ*cps* accumulated significantly more l-histidine in repeated cultivations compared to *C. glutamicum* CgHis2 (Table 2, Fig. 4).

**Table 2:** Constructed *C. glutamicum* CgHis2 strains with individual gene deletions and the respective l-histidine titer obtained in microtiter plate-scale cultivations

*C. glutamicum* strain	Locus tag	Protein/enzyme	His-Titer [mM]	His-Titer [%]	Biomass [final OD_600_]
CgHis2 reference			11,5 ± 0.6	-	19.3 ± 0.5
CgHis2 *ΔfasB*	NCgl2409	Fatty acid synthase	14.3 ± 0.4	+ 25	18.4 ± 0.7
CgHis2 *ΔNCgl2964*	NCgl2964	DEAD/DEAH box helicase	10.6 ± 0.3	− 8	18.5 ± 0.2
CgHis2 *ΔfasA*	NCgl0802	Fatty acid synthase	No deletion possible		
CgHis2 *Δpks*	NCgl2773	Type 1 polyketide synthase	No deletion possible		
CgHis2 *Δemb*	NCgl0184	Arabinosyl transferase C	No transformation of pSenHis[*hisEG*] possible		
CgHis2 *ΔgltB*	NCgl0181	Alpha subunit of glutamate synthase	11.6 ± 0.2	0	21.9 ± 0.1
CgHis2 *ΔNCgl0552*	NCgl0552	DNA segregation ATPase	11.1 ± 0.6	− 4	19.6 ± 0.6
CgHis2 *Δcps*	NCgl2618	Non-ribosomal peptide synthase	13.6 ± 0.2	+ 18	18.6 ± 2.3
CgHis2 *ΔpknB*	NCgl0040	Eukaryotic-type serine/threonine kinase	No deletion possible		
CgHis2 *ΔNCgl2959*	NCgl2959	Phosphoesterase	10.9 ± 0.2	− 6	19.4 ± 0.4
CgHis2 *ΔNCgl1737*	NCgl1737	Hypothetical membrane protein	10.8 ± 0.3	− 8	18.8 ± 0.2
CgHis2 *ΔNCgl2981*	NCgl2981	Hypothetical protein	No deletion possible		
CgHis2 *ΔggtB*	NCgl0916	Putative gamma-glutamyltranspeptidase precursor PR	10.3 ± 0.4	− 12	20.0 ± 0.4
CgHis2 *ΔNCgl0705*	NCgl0705	Probable ATP-dependent helicase	11.8 ± 0.5	+ 2	18.8 ± 0.5
CgHis2 *ΔputA*	NCgl0098	Proline dehydrogenase/delta-1-pyrroline-5-carboxylate dehydrogenase	9.7 ± 0.4	− 17	20.3 ± 0.5
CgHis2 *Δpyk2*	NCgl2809	Pyruvate kinase	10.6 ± 0.3	− 8	18.1 ± 0.4
CgHis2 *Δpyk1*	NCgl2008	Pyruvate kinase	12.1 ± 0.4	+ 5	21.5 ± 1.0

The fatty acid synthase FasB/FAS-IB is a non-essential, primarily palmitate-synthesizing polyketide synthase encoded by *fasB* (*NCgl2409*) in *C. glutamicum* ATCC 13032 [41]. During FAAMS-analysis, *fasB* turned out as the most interesting gene potentially contributing to increased l-histidine formation as it harbored SNPs in 24% of all *C. glutamicum* CgHis2 strain variants independently isolated by FACS (Table 1). Whereas the individual reintroduction of two of these SNPs (G1921E, G2762D) showed no beneficial effect on product synthesis, *C. glutamicum* CgHis2 *ΔfasB* was characterized by a significant increase of the l-histidine titer by 25%. The pyruvate kinase, encoded by *pyk1* (*NCgl2008*), is a key enzyme in regulation of the glycolytic flux in response to the cell’s energy state [42, 43]. More recently, a second functional *pyk* gene (*pyk2*, *NCgl2809*) was identified, but only found to be highly transcribed under oxygen-deprived conditions [44]. Interestingly, we identified *pyk2* during the comparative genome analysis only as a potential hotspot gene of medium significance (in 11% of the isolated *C. glutamicum* CgHis2 variants). The investigated point mutation *pyk2*-T357I and a *pyk2* in-frame deletion in *C. glutamicum* CgHis2 did not result in an increased l-histidine synthesis. Only an additionally constructed *C. glutamicum* CgHis2 *Δpyk1* variant (a gene for which only four mutations in four different variants could be identified) showed increased l-histidine production capabilities over repeated cultivations, even though (according to Student’s *t* test) the 5% increase in l-histidine titer was not significant (*p* = 0.096). Deletion of the *cps*-gene increased l-histidine production by 18% and hence outperformed the previously investigated *C.* *glutamicum* CgHis2 *cps*-G987D variant (12%). Unfortunately, the attempted deletion of *pks* and *NCgl2981* in *C. glutamicum* CgHis2 did not yield any variants with correct in-frame deletion.

### Combination of beneficial mutations boosts l-histidine production

Subsequently, beneficial genomic mutations and gene deletions were step-wise combined to benefit from potentially additive effects on product formation. Four *C. glutamicum* CgHis2 variants with two genomic modifications showed an increased l-histidine formation. In this context, the *fasB* deletion turned out the to be a key engineering target as the combination of this modification with the deletion of *pyk1* or *cps*, or with the identified SNP in the *pks*-gene (*pks*-D1186N) increased the product formation of 29, 40, and 30%, respectively (Fig. 4). Interestingly, only the constructed double mutant *C. glutamicum* CgHis2 *Δpyk1 NCgl2981*-D735G outperformed the respective single mutant strains as well (Additional file 1: Fig. S8).

Next, the best-performing double mutant *C. glutamicum* CgHis2 Δ*fasb* Δ*cps* served as starting variant for the construction of various triple mutants (*C. glutamicum* CgHis2Δ*fasB* Δ*cps* Δ*pyk1*, *C. glutamicum* CgHis2Δ*fasB* Δ*cps* *pks*-D1186N, *C. glutamicum* CgHis2 Δ*fasB* Δ*cps NCgl2981*-D735G), quadruple mutants (*C. glutamicum* CgHis2 Δ*fasB* Δ*cps* Δ*pyk1* *pks*-D1186N, *C. glutamicum* CgHis2 Δ*fasB* Δ*cps* Δ*pyk1* *NCgl2981*-D735G), and a quintuple mutant (*C. glutamicum* CgHis2 Δ*fasB* Δ*cps* Δ*pyk1* *NCgl2981*-D735G *pks*-D1186N). In this strain lineage, however, no further improvements in l-histidine production but strongly reduced growth performance of the constructed variants could be observed (Additional file 1: Figure S9). However, *C. glutamicum* CgHis2 Δ*fasb* Δ*pyk1* turned out as more suitable starting variant. In combination with the *NCgl2981*-D735G and the *pks*-D1186N mutation, the quadruple mutant *C. glutamicum* CgHis2 Δ*fasB* Δ*pyk1* *NCgl2981*-D735G *pks*-D1186N proved to be the best-performing l-histidine producer. This variant accumulated up to 18.7 mM l-histidine, accounting for a 69% increase in direct comparison to *C. glutamicum* CgHis2 within 48 h. At the same time, however, the biomass formation was reduced by 21%.

### Lab-scale bioreactor cultivations of selected variants

**Fig. 5:**
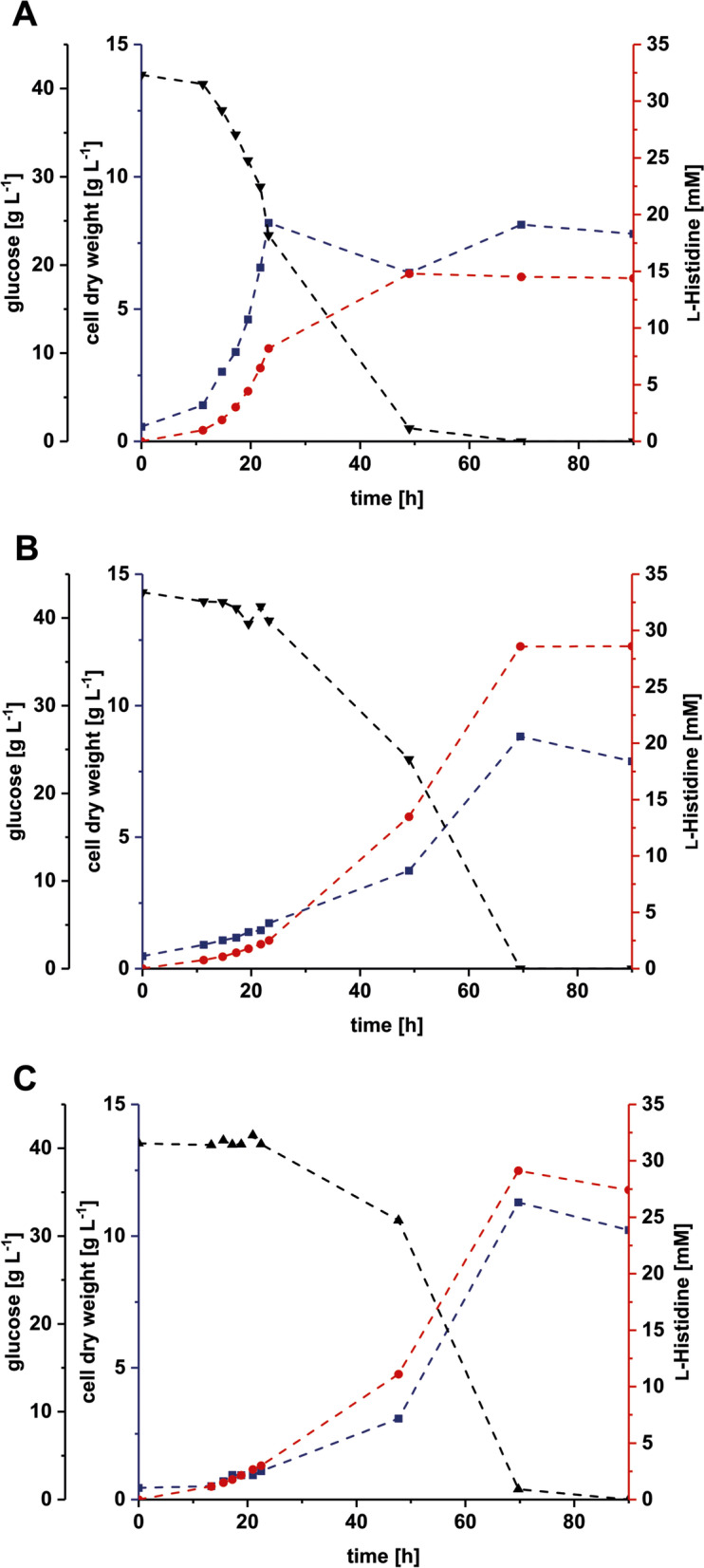
Lab-scale batch cultivations of **A** the *C. glutamicum* CgHis2 reference strain, **B** the FACS-isolated *C. glutamicum* CgHis2 12–10-5–6 variant, and **C** the reverse engineered *C. glutamicum* CgHis2 Δ*fasB* Δ*pyk1 NCgl2981*-D735G *pks*-D1186N**.** Bioreactor cultivations were performed in duplicates using defined CGXII medium with 40 g L^*−*1^ d*-*glucose as sole source of carbon and energy. Data points represent the average of two independent bioreactor cultivations

With the aim to investigate the suitability of selected l-histidine production strains for biotechnological applications, bioreactor batch fermentations were performed at laboratory scale. In these experiments, the performance of the starting strain *C. glutamicum* CgHis2 was compared to the rationally engineered quadruple mutant *C. glutamicum* CgHis2 Δ*fasB* Δ*pyk1* *NCgl2981*-D735G *pks*-D1186N and the randomly mutated *C. glutamicum* CgHis2 12–10-5–6 variant (Fig. 5), which was the best-performing FACS-isolated variant bearing 120 SNPs and accumulating 16.8 ± 0.4 mM l-histidine in microtiter cultivations. From bioprocess modeling, maximum specific rates for biomass growth, d-glucose uptake, and l-histidine production were derived (Table 3).

**Table 3:** Key Performance Indicators of selected *C. glutamicum* CgHis2 variants obtained from lab-scale bioreactor cultivations in batch mode. Maximum titers and yields were derived directly from measurements and specific rates via process modeling. For the latter, asymmetric confidence bounds were estimated by following a parametric bootstrapping approach (values in brackets)

Strain	CgHis2 reference	CgHis2 variant 12–10-5–6	CgHis2 Δ*fasB* Δ*pyk1 NCgl2981*-D735G *pks*-D1186N
OD_600_ [-]	30.4	30.6	41.5
CDW [g L^−1^]	8.3	8.8	11.3
l-histidine titer [mM]	14.8	28.6	29.1
Volumetric productivity [g L^−1^ h^−1^]	0.03	0.05	0.05
l-histidine yield [C-mol C-mol^−1^]	0.07	0.13	0.13
Growth rate [h^−1^]	0.15 [0.148, 0.160]	0.06 [0.054, 0.058]	0.05 [0.048, 0.052]
d-glucose uptake rate [mmol g_CDW_ h^−1^]	1.96 [1.768, 2.139]	1.28 [1.167, 1.383]	1.07 [0.995, 1.137]
l-histidine production rate [mmol g_CDW_ h^−1^]	0.13 [0.122, 0.142]	0.14 [0.131, 0.154]	0.17 [0.157, 0.188]

In these experiments, the FACS-isolated variant *C. glutamicum* CgHis2 12–10-5–6 as well as the reverse engineered *C. glutamicum* CgHis2 Δ*fasB* Δ*pyk1* *NCgl2981*-D735G *pks*-D1186N produced about twice as much l-histidine as the *C. glutamicum* CgHis2 starting strain (28.6, 29.1 and 14.8 mM, respectively; Fig. 5). Even though both variants grew with significantly reduced growth rates and d-glucose uptake rates, the reverse engineered variant formed significantly more biomass (11.3 g L^−1^ CDW) compared to the FACS-isolated mutant and the *C. glutamicum* CgHis2 starting strain, which showed a similar biomass formation (8.8 and 8.3 g L^−1^ CDW, respectively). However, both advanced *C. glutamicum* CgHis2 variants were characterized by an almost doubled substrate-dependent product yield, which increased from 0.07 to 0.13 C-mol C-mol^−1^.

## Discussion

Directed evolution of microorganisms offers the possibility to identify beneficial alterations of enzymes, cellular structures, or genetic regulation in the context of product formation, of which most would not have been considered (or could not have been harnessed) during rational metabolic engineering [45]. However, the complexity of the microbial metabolism typically requires the screening of very large and genetically diverse strain libraries, which should be accomplishable within a reasonably short time frame and at low costs — something biosensor-based high-throughput screening strategies involving FACS can offer [46]. In case identification of underlying beneficial mutations is desired, automated comparative and combinatorial genome analysis on the basis of multiple genomes and reverse engineering has to be also performed [47–50].

In this study, we set out to improve the product formation of an industrial l-histidine producing *C. glutamicum* variant, which already bears multiple genetic modifications in known targets contributing to l-histidine accumulation. This strain converts d-glucose to l-histidine with a product yield of 0.07 C-mol C-mol^−1^ (0.06 g l-histidine g d-glucose^−1^) and still performs far from the theoretical maximum product yield of 0.44 g l-histidine g d-glucose^−1^ (at a growth rate of 0.1 h^−1^) [6].

Previous random or rational engineering studies mostly investigated the role of the feedback-inhibited and rate-limiting ATP phosphoribosyltransferase (HisG) as key enzyme of the l-histidine biosynthesis pathway [11, 24, 25, 27]. Several beneficial *hisG* mutations were discovered by these approaches. Hence, the l-histidine production strain used as starting variant in this study, harbored the *hisG*_S143F-ΔC_ gene encoding for a feedback-deregulated HisG variant to increase the flux into the l-histidine pathway [27]. Interestingly, additional unknown mutations in *hisG* were found in 9% of isolated *C. glutamicum* CgHis2 variants in this study, but these mutations were not further investigated due to the focus on identifying novel targets contributing to l-histidine production. Low mutation frequency in other l-histidine biosynthesis genes, already overexpressed in *C. glutamicum* CgHis2, indicated this engineering module to be majorly exhausted. The same is true for known genetic targets involved in purine biosynthesis such as *purA*, *purB*, and *purH* [6, 20, 22, 37]. Overexpression of a heterologous glycine cleavage system was previously reported to be beneficial for l-histidine synthesis since an improved C_1_-supply increases conversion of pathway intermediate AICAR to ATP biosynthesis [6]. No such system is present in *C. glutamicum* CgHis2, but *glyA*, encoding the endogenous C_1_- unit supplying serine hydroxymethyltransferase, was found to be only mutated in three isolated variants. In conclusion, selection pressure on known engineering targets appeared to be low in *C. glutamicum* CgHis2 such that this rationally engineered l-histidine-producing variant could be successfully leveraged for the identification of additional beneficial off-site mutations.

By using automated comparative and combinatorial genome analysis, over 18,000 randomly induced mutations were analyzed regarding their hotspot significance, hotspot abundance, respective strain variant performance, and hypothesized metabolic network effects, even though the strong mutational bias of MNNG towards GC→AT transition mutations could be confirmed (Additional file 1: Fig. S6) [51–53].

Three SNPs contributing to increased l-histidine accumulation in *C. glutamicum* CgHis2 could be reliably identified in three different genes (*cps, pks, NCgl2981*). Noteworthy, the metabolic roles of *cps* and *NCgl2981* in *C. glutamicum* are unknown, and neither of the two has been considered for any strain engineering purposes yet. However, only *cps*-G987D could be verified as loss-of-function mutation, since the in-frame deletion of *cps* had a greater beneficial effect on l-histidine production in *C. glutamicum* CgHis2.

The *cps*-gene encodes for a putative non-ribosomal peptide synthetase (NRPS). These large enzymes are typically involved in the synthesis of cyclic or branched peptides containing non-proteinogenic amino acids. The peptides are considered as secondary metabolites and can serve as antibiotics, siderophores, or pigments [54]. To the best of our knowledge, no role has been assigned to *cps* yet, and no non-ribosomal peptide has been described in *C. glutamicum* to this day. However, typically an NRPS initially catalyzes the ATP-consuming activation of an amino acid via adenylation. Decreasing ATP consumption by a less active Cps might explain the beneficial effect of the *cps*-mutation on l-histidine synthesis. Schwentner and co-workers identified a high ATP regeneration capacity necessary for efficient l-histidine production by performing Flux Balance Analysis [6]. This may account for preventing ATP hydrolysis as well. Interestingly, several other genes encoding ATP-dependent enzymes such as helicases (*NCgl2964*, *NCgl0302*, *NCgl0705*, *NCgl2433*), ATPase components (*NCgl0552*, *NCgl2859*, *NCgl1085*) and a protease (*clpC*) were identified as potential hot spots. In closely related mycobacteria, such as *Mycobacterium smegmatis* and *Mycobacterium abscessus*, NRPS are involved in synthesis of cell wall components with the respective genes located in direct proximity to other cell-wall-related genes [55]. In *C. glutamicum*, a MarR-type regulator gene is located directly upstream of *cps* (Additional file 1: Fig. S10). Even though its position and *cps* itself are highly conserved in many *Corynebacterium* species, we could not find any significant regulation of this gene in the compendium of 927 microarray-based transcriptional profiles available for *C. glutamicum* ATCC 13032 [56]. However, modulation of the putative NRPS activity may also benefit l-histidine production by an alteration of the cell wall composition.

*NCgl2981* encodes a hypothetical protein of yet unknown function or structure. Its annotation as hypothetical membrane protein is only supported by a single transmembrane helix predicted by TMHMM 2.0 [57]. The sequence of *NCgl2981* and its genomic position is highly conserved among various *Corynebacterium* and *Mycobacterium* species (Additional file 1: Fig. S11). Located upstream is *mutT*, potentially involved in DNA repair, and in close proximity a *N*-acetylmuramoyl-l-alanine amidase encoding gene, which is involved in cell wall synthesis (ERGO systems biology informatics toolkit, Igenbio Inc., Chicago, IL, USA). In the compendium of expression profile data, we found no significant regulation of this gene [56]. According to the AlphaFold structure prediction, the identified *NCgl2981*-D735G substitution is located in a loop on the protein surface close to a C-terminal transmembrane helix (Additional file 1: Fig. S7) [58]. Since a *C. glutamicum* CgHis2 variant with a deletion of *NCgl2981* could not be generated, it can be only assumed that NCgl2981 is involved in essential cellular processes.

The polyketide synthase gene *pks* is essential for mycolic acid synthesis [38, 39]. *C. glutamicum* Δ*pks* strains are mycolic acid-deficient and therefore exhibit an altered cell envelope [59]. As a result, more resources might become available for l-histidine synthesis. Alternatively, the excretion of l-histidine could be increased due to the altered cell envelope as it was also observed in the case of the l-lysine producing *C. glutamicum* ATCC 21527 variant with a reduced mycolic acid content [60, 61]. Hence, *pks*-D1186N may represent a beneficial loss-of-function mutation, even though we were unsuccessful in deleting *pks* in the already extensively engineered *C.* *glutamicum* CgHis2 variant.

With *fasA* and *fasB*, we identified two significant hotspot genes in *C. glutamicum* CgHis2, which supply C_18_ oleic acid and C_16_ palmitic acid precursors for cell wall synthesis in *C. glutamicum*, respectively [39, 41]. Since FasA-activity is essential for growth, no *fasA*-deletion was attempted in the *C. glutamicum* CgHis2 strain background as oleic acid supplementation is unfeasible for any industrial l-histidine production [62]. FasB on the other hand is dispensable for growth, and previous studies show that *C. glutamicum ΔfasB* variants are characterized by an altered phospholipid- and mycolic acid profile [41]. Hence, more available resources and/or altered cell wall structures may benefit l-histidine synthesis and/or l-histidine efflux in the isolated (and reconstructed) *C. glutamicum* CgHis2 *fasB* variants. In addition, a proposed metabolic connection in *C. glutamicum* between FasB activity and the C_1_ metabolism via the cofactor lipoic acid may affect l-histidine biosynthesis, too [6, 62–64]. Moreover, *fasB* deletion was reported to exert a significant beneficial effect on l-glutamate production in a *C. glutamicum* *ΔfasAB* double mutant [41]. Interestingly, we found 41% of CgHis2 strain variants with nonsynonymous mutations in *fasA* to also harbor nonsynonymous mutations in *fasB*. The significantly increased l-histidine production of isolated CgHis2 variant 12–10-5–6 may have been majorly attributed to its acquired *fasAB* mutations. However, several attempts to construct *fasAB* double mutants in CgHis2 failed.

*C. glutamicum* deletion mutants of *pyk1*, encoding a pyruvate kinase, were previously reported to be characterized by an increased d-glucose uptake, increased l-glutamate production, and increased pools of pentose phosphate pathway metabolites [42, 43]. We deleted *pyk1* in *C. glutamicum* CgHis2 since deletion of the second pyruvate kinase gene *pyk2*, which was originally identified as part of this study did not show beneficial effects on l-histidine production. Since *pyk2* is highly transcribed under oxygen-deprived conditions, typically not given during l-histidine production, we considered Pyk1 to be more likely to contribute to l-histidine production due to its link to ATP metabolism and its key function in the regulation of glycolytic flux [42–44]. Hence, this led to increased l-histidine production titers in CgHis2 ∆*pyk*1 mutants, even though the ATP-generating function of *pyk1* should be eliminated. This may be attributed to a reduction of glycolytic flux and increase of pentose phosphate pathway flux towards l-histidine.

Taken together, several novel genetic targets with no obvious connection to l-histidine biosynthesis, but significant contribution to l-histidine production with *C. glutamicum* CgHis2, could be identified in the conducted biosensor-based FACS screening. Interestingly, three large genes (*cps*, 3,885 bp; *pks*, 4,830 bp; *fasB*, 8,988 bp) could be identified, whose in-frame deletion resulted in significant contribution to an increased l-histidine production. Hence, an initial hypothesis was that the respective beneficial effects could be also attributed to a simple genome reduction. Genome reduction strategies aim at improving production processes by removing non-essential genes and enzyme machineries to save resources (nucleoside triphosphates, amino acids, ATP, etc.), which would then become available for product formation [65–67]. In a previous study however, deletion of much larger non-essential gene clusters in *C.* *glutamicum* did not increase l-lysine product titers [68]. Furthermore, one could argue that larger genes are more likely to be identified in the mutagenesis/screening strategy presented here, because larger genes would be more frequently mutagenized during random chemical mutagenesis. However, in our experiments, we found that the three genes in question were mutated more frequently than would have expected considering their respective sequence length (*cps*, 1.7-fold; *pks*, 1.6-fold; *fasB*, 1.1-fold).

In subsequent experiments, SNPs and gene deletions were combined to identify additive effects of individual mutations. Here, the combination of the identified point mutations in *pks* and *NCgl2981* (*pks*-D1186N and *NCgl2981*-D735G) and deletion of *fasB* and *pyk1* turned out to be the best-performing l-histidine producing *C. glutamicum* CgHis2 variant. Even though no fed-batch mode was performed in the bioreactor experiments, the product yield (0.13 mol l-histidine mol d-glucose^−1^ or 0.11 g l-histidine g d-glucose^−1^) and product titer (4.5 g l-histidine L^−1^) could be further improved in comparison to earlier studies in *C. glutamicum* [6, 26]. Interestingly, this reengineered quadruple mutant performed very similar to the *C. glutamicum* CgHis2 12–10-5–6 variant, which was characterized by the highest l-histidine titer among the 100 FACS-isolated *C. glutamicum* CgHis2 mutants in this study. Both variants accumulated twice as much l-histidine compared to the starting variant and are characterized by a similar growth rate. This raises the question whether the in-depth characterization and comparative genome analysis of all variants, along with the laborious combinatorial reconstruction of many strain variants, was necessary. From an industrial perspective, many microbial production systems used at industrial scale are not fully understood yet [4]. However, more robustness is considered for industrial strains when detrimental mutations acquired from random mutagenesis are omitted by reverse engineering [49]. In addition, higher biomass formation and an increased production rate should render this strain more suitable for industrial scale-up [69]. From an academic standpoint, the identification of hitherto unknown genes with a link to the production of a small metabolite might promote further research. Furthermore, novel genomic targets for metabolic engineering with a positive but product-unspecific effect such as genes involved in cell wall synthesis might also be useful for the development of microbial production strains for a multitude of other valuable small metabolites.

## Conclusions

The identification of novel genomic targets for metabolic engineering remains a challenging task, but the high-throughput strategies presented here might serve as a blueprint for successful future campaigns. In particular, when already highly engineered production strains are to be further improved and all known metabolic engineering targets are exhausted. When suitable biosensors are at hand or can be engineered to detect the desired metabolite, high-throughput screening and automated genome analysis can deliver valuable, large datasets within a limited time frame. In the future, advanced machine learning tools will allow for clearer predictions, not only during the identification of useful targets, but also when several novel targets need to be combined to be able to push microbial production strains towards maximum performance.

## Methods

### Construction of plasmids and strains

All bacterial strains and plasmids used in this study are provided in Additional file 1: Table S1. *E. coli* DH5α was used for recombinant DNA work und grown in LB medium at 37 °C with 50 µg mL^−1^ kanamycin when appropriate [70]. Standard protocols of molecular cloning such as PCR, DNA restriction, and ligation were followed for construction of plasmids [71, 72]. Transformation of *C. glutamicum* was performed by electroporation as described earlier [73]. Introduction of point mutations and in-frame gene deletions in the genome of *C. glutamicum* was performed by double homologous recombination using the pK19*mobsacB* vector system [74]. All oligonucleotides used were ordered from Eurofins Genomics Germany GmbH (Ebersberg, Germany) and are listed in Additional file 1: Table S2. Sanger Sequencing was performed at Eurofins Genomics GmbH as well.

### Cultivation of *C. glutamicum* strains

*C. glutamicum* was routinely cultivated at 30 °C in Brain–Heart-Infusion (BHI) medium (Difco Laboratories, Detroit, MI, USA) or in defined CGXII medium using 2% d-glucose as sole source of carbon and energy [75]. Where appropriate, 15 µg mL^−1^ kanamycin was added. In general, biomass formation was followed by measuring the optical density at 600 nm (OD_600_). For dipeptide supplementation experiments, the BioLector cultivation system (m2p-labs GmbH, Baesweiler, Germany) with 48-well microtiter flowerplates was used to monitor formation of biomass and fluorescence during the cultivation of biosensor-harboring strains (30 °C, 900 rpm, 75% humidity, and 3 mm throw for 48 h). Information on biomass formation was provided as backscattered light at 620 nm by the BioLector, whereas this device measures EYFP fluorescence emission at 532 nm after excitation at 510 nm. The seedtrain comprised stationary BHI and CGXII (2% d-glucose) precultures to inoculate the main culture (CGXII, 2% d-glucose) to an OD_600_ of 1. Dipeptides were purchased from Bachem (Bubendorf, Switzerland) and added to the main culture medium before inoculation.

For high-throughput characterization of the *C. glutamicum* CgHis2 strain variants, a two-step workflow comprising two subsequent cultivations with product analysis via HPLC was developed. Both cultivation steps were performed in 48-well microtiter Flowerplates (m2p-labs GmbH, Baesweiler, Germany) in a Multitron Pro HT Incubator (InforsAG, Bottmingen, Switzerland, at 30 °C, 900 rpm, 75% humidity, and 3 mm throw for 48 h). For high-throughput and high accuracy, a Hamilton Starlet liquid handling platform (Hamilton, Reno, NV, USA) was used for sample preparation of main cultures. Biomass formation was determined as final OD_600_ after 48 h of cultivation using a Synergy Mx microplate reader (BioTek, Winooski, VT, USA). In cultivation 1, CGXII main cultures were inoculated from BHI precultures (incubated for 22 h) using no technical replicates. l-histidine titers exceeding the 90% confidence interval of those of the control strains (with 4 biological and 3 technical replicates each) were regarded as preliminary significant. Additionally, a threshold of 1 mM increase in product titer was set as threshold for variants to be considered for the second, in-depth characterization step. In this second step, all preselected strain variants were cultivated in technical triplicates starting from BHI precultures (incubated for 22 h) and subsequent CGXII precultures (2% d-glucose). To confirm the l-histidine-overproducing phenotype, strain variants had to exceed the 90% confidence interval of control strains. Reverse engineered strains were characterized according to the second characterization step.

### Amino acid quantification

For quantification of amino acids, culture supernatants were diluted using a Hamilton robotic liquid handling platform (Hamilton, Reno, NV, USA). High-performance liquid chromatography was performed using an uHPLC 1290 Infinity system (Agilent Technologies, Santa Clara, CA, USA) equipped with a Zorbax Eclipse AAA C18 3.5 micron 4.6 × 75 mm and a fluorescence detector. Amino acids were quantified by their *O*-phthaldialdehyde derivatives [76]. A gradient of 0.01 M Na-borate buffer pH 8.2 with increasing concentrations of methanol was used as mobile phase. Fluorescence detection of the isoindole derivatives was performed at an excitation wavelength of 230 nm and an emission wavelength of 450 nm.

### Multiplexed MNNG mutagenesis

Multiplexed chemical mutagenesis using the mutagenic agent *N*-methyl-*N'*-nitro-*N*-nitrosoguanidine (MNNG) was applied to generate independently mutagenized batches for FACS screening [53]. In order to generate cultures with a desired mortality of 50–90%, varying MNNG concentrations were used for 96-well plate mutagenesis experiments, as follows:

Fifty-microliter BHISG (BHI medium supplemented with 91 g L^−1^ sorbitol, 1% d-glucose, and 15 μg mL^−1^ kanamycin) precultures were grown overnight to inoculate 50 mL BHISG main cultures to an OD_600_ of 1.5. After incubation for 2–3 h in a 30 °C rotary shaker at 120 rpm (Infors, Bottmingen, Switzerland), at an OD_600_ of 4.4, the cultures were immediately transferred to a 2-mL 96-well plate (Eppendorf, Hamburg, Germany) by splitting into 96 × 400 μL aliquots. For mutagenesis, a 1–5 mg mL^−1^ MNNG-in-DMSO series was prepared. Twenty microliters of the respective solution was applied to each well of columns 4–12 with descending MNNG concentrations in rows A–H. Columns 1–3 were treated with DMSO only and served as respective mutagenesis controls for subsequent FACS screenings. Mutagenesis was performed in a rotary shaker at 30 °C and 120 rpm for exactly 15 min (Infors, Bottmingen, Switzerland). Mutagenesis was stopped by centrifugation in a tabletop centrifuge (Heraeus Multifuge X3R Centrifuge, Thermo Fisher Scientific Inc., Waltham, MA, USA) at 20 °C and 4000 rpm for 7 min and two subsequent washing steps of the cultures using 1.6 mL of a 0.9% NaCl solution, after which the cultures were regenerated in 400 μL BHISG for 3 h. Subsequently, single-cell FACS sorting of the main population was performed to evaluate the culture mortality of representative mutagenesis batches of every MNNG condition. For storage until FACS screening, 400 μL 80% glycerol were added and the mutagenized cultures were kept at − 80 °C.

### FACS screening

Prior to FACS screening, 400 µL of the mutagenized cultures and control cultures of *C. glutamicum* CgHis2 were used to inoculate 800 µL of defined CGXII medium (2% d-glucose, 15 µg mL^−1^ kanamycin) each. The cultivations were performed in 48-well microtiter Flowerplates (m2p-labs GmbH, Baesweiler, Germany) in a Multitron Pro HT Incubator (Infors AG, Bottmingen, Switzerland, at 30 °C, 900 rpm, 75% humidity, and 3 mm throw) for 6 h. For analysis of single-cell size and fluorescence, a BD FACSAria Fusion Flow Cytometer (BD Biosciences, Franklin Lakes, NJ, USA) was used. Cultures were diluted in CGXII base medium to obtain 10,000 events s^−1^ at a flow rate of 1.0 AU when using a 70-µm nozzle at a sheath pressure of 70 psi. A 488-nm blue solid laser was used for excitation of eYFP. Forward scatter (FSC, as small angle scatter) and side scatter (SSC, as orthogonal scatter) of the 488-nm laser were used to record the single-cell FSC, SSC, and fluorescence characteristics. Therefore, a 502-nm long-pass and 530/30 band-pass filter combination was employed. Electronic gating of FSC-H against SSC-H was used to minimize electronic noise and cell debris. Subsequent gating of SSC-H against SSC-W and FSC-H against FSC-W was performed for doublet discrimination. Fluorescence and FSC analysis of *C. glutamicum* populations were always performed using this gating scheme. For cell sorting of each mutagenized culture, the top 5% of fluorescent cells in relation to the FSC distribution (as cell size parameter) were gated and subsequently 240 events were sorted on kanamycin-containing agar plates in single-cell mode. Control cultures were used to compare the MNNG-induced fluorescence heterogeneity and as technical cell sorting controls. After 2 days incubation at 30 °C, 22 strain variants per mutagenesis batch were randomly picked from these agar plates and transferred to 600 µL BHISG (BHI medium supplemented with 91 g L^−1^ sorbitol, 1% d-glucose, and 15 μg mL^−1^ kanamycin) in 96-well deep-well microtiter plates (Eppendorf SE, Hamburg, Germany). These plates were cultivated in a Multitron Pro HT Incubator (Infors AG, Bottmingen, Switzerland, at 30 °C, 900 rpm, 75% humidity, and 3 mm throw) for 24 h. After addition of 400 µL 85% glycerol, the isolated strain variants were stored at − 80 °C.

### Whole genome sequencing

The isolation of chromosomal DNA from *C. glutamicum* was performed according to established protocols [77]. Whole genome sequencing was performed at Eurofins Genomics Germany GmbH (Ebersberg, Germany) on an Illumnia High-Seq in paired-end mode using 150 bp read length. The genome sequencing raw data of the 100 biosensor/FACS-isolated *C. glutamicum* variants are stored at Sequence Read Archive (SRA) of the National Library of Medicine (NCBI) and can be found under the BioProject ID PRJNA990379 [34].

### Fast Automated Analysis of Multiple Sequences—FAAMS

The whole genome sequencing analysis for the high number of sequenced clones was performed using an automated workflow. This workflow includes downloading reference data for *C. glutamicum* ATCC 13032 from NCBI via the NCBI-Genome-Download tool (https://github.com/kblin/ncbi-genome-download). First, fastq files of the sequenced 100 variants obtained from the Eurofins Genomics Germany GmbH are each pre-processed (e.g., trimming, filtering of bad reads) with fastp and subsequently aligned to the downloaded wild-type reference with the Burrows-Wheeler Aligner, BWA [78, 79]. Hereby, the BWA-MEM algorithm was used. Picard toolkit (Broad Institute, 2019) and Samtools were used to retrieve statistical data for quality control while the latter was also used for pileup before identification of variants (SNP and InDel) by VarScan [80–82]. Sequences of CgHis2 strain variants were compared to the starting strain sequence. Differences to the starting strain were annotated on the gene level (codon/amino acid) while deviations up to 200 bp upstream of a gene were labeled as potential mutations in the promoter region. Statistical analysis including quality control of sequence reads and alignments was automatically performed by R. Data output was visualized in a customized R shiny dashboard [83].

Computational analysis of FAAMS data was performed in Python 3.8 using standard data analysis and visualization libraries. The corresponding Jupyter notebook for FAAMS data processing is provided on GitHub (https://github.com/JuBiotech/Supplement-to-Baumann-et-al.-BeyondRational-2022).

### Lab-scale bioreactor cultivations

The seedtrain for the bioreactor cultivations comprised two precultures before inoculation of the bioreactor. The first preculture (10 mL BHI in 100 mL shaking flask with baffles) was inoculated from a fresh agar plate. The culture was incubated at 30 °C and 250 rpm on a rotary shaker (Infors, Bottmingen, Switzerland) for 18–38 h. Five milliliters of the first preculture were used to inoculate the second preculture (100 mL CGXII, 2% d-glucose in a 1-L shake flask), which was cultivated in accordance to the first preculture for 18–28 h. The stationary cultures were centrifuged and washed using 0.9% NaCl to a final volume of 5 mL. An appropriately diluted aliquot was transferred to the bioreactor to inoculate the main culture (988 mL CGXII, 2% d-glucose, 2 mL antifoam, 10 mL inoculum) to an OD_600_ of 0.5. Kanamycin was added to all cultivations appropriately.

The bioreactor fermentation was performed on a DASGIP system (Eppendorf SE, Hamburg Germany) with four simultaneously operated reactors. Regulation of pH was performed both-sided with 5 M H_2_PO_4_ and 25% NH_4_OH. Dissolved oxygen was fixed via a cascade to a minimum of 30% by increasing agitation from 400 to 1200 rpm. Hence, the airflow increased from 6 to 40 standard liter h^−1^ when needed. The fermentation was stopped when the optical density had surpassed its peak and agitation had returned to 400 rpm, which meant the carbon source was depleted. During the fermentation, a series of 6-mL samples were taken from the cultures for determination of d-glucose and l-histidine concentrations as well as OD_600_ and cell dry weight.

d-glucose measurements were performed on an Agilent 1260 Infinity II HPLC System (Santa Clara, CA, USA) equipped with a 300 × 8 mm Organic Acid Column (Chromatographie Service GmbH, Langerwehe, Germany) with a up to fourfold dilution. Cell dry weight was determined from 1.8-mL samples, washed in 0.9% NaCl, and dried at 80 °C for at least 1 day with subsequent incubation in a desiccator for 1 day. The weight was determined according to pre-weighed tubes on a precision scale.

### Bioprocess modeling

Data from all bioreactor experiments were modeled using classical Monod kinetics to describe biomass growth, d-glucose consumption, and l-histidine formation. Some model parameters such as Monod constants and specific yields were introduced as global parameters along the three batches, while all others were estimated separately as local parameters. For model implementation, validation, and analysis, we used the open-source, python-based modeling tool pyFOOMB [84]. Details on the models and fitting results can be found in the corresponding Jupyter notebook provided on GitHub (https://github.com/JuBiotech/Supplement-to-Baumann-et-al.-BeyondRational-2022).

### Supplementary Information

**Additional file 1: Figure S1**. Comparison of *C. glutamicum* CgHis1 pHisOP1 and its variant *C. glutamicum* CgHis2, which served as screening host. **Figure S2.** Specific fluorescence response of biosensor-harboring *C. glutamicum* strains upon initial dipeptide supplementation**. Figure S3.** Biosensor crosstalk – Co-cultivation of *C. glutamicum* CgHis2 and *C. glutamicum* wild type pSenHis. **Figure S4.** Schematic overview of the screening workflow for the identification of significantly improved l-histidine producing *C. glutamicum* CgHis2 strain variants. **Figure S5.** Individual SNP-distribution across the genome of all 100 FACS-isolated and l -histidine producing *C. glutamicum* CgHis2 variants. **Figure S6.** Mutational bias of MNNG-mutagenesis. **Figure S7.** Predicted structure of NCgl2981 and position of the D735 residue as calculated by AlphaFold. **Figure S8.** l-histidine production performance of reverse engineered CgHis2 single mutant- and double mutant strains. **Figure S9.** l-histidine production performance of reverse engineered CgHis2 variants in comparison to the CgHis2 reference strain. **Figure S10.** Genomic position of the *cps*-gene encoding the non-ribosomal peptide synthase with an unknown MarR-type regulator gene located directly upstream in *C. glutamicum* ATCC 13032. The position of the *cps*-gene and the gene of the MarR-type regulator gene are highly conserved among various *Corynebacterium* species. **Figure S11.**Genomic position of the *NCgl2981* gene of unknown function in *C. glutamicum* ATCC 13032. *NCgl2981* is highly conserved among various *Corynebacterium* and *Mycobacterium* species. **Table S1.** Bacterial strains and plasmids. **Table S2.** Oligonucleotides. **Table S3.** Identified mutations in already known genetic targets contributing to l-histidine production from 100 improved *C. glutamicum* CgHis2 variants. **Table S4.** Hotspot genes in *C. glutamicum* CgHis2 identified by computational analysis of the FAAMS dataset. **Table S5.** List of SNPs identified in 100 independently isolated l-histidine producing *C. glutamicum* CgHis2 variants, selected for individual reconstruction in the *C. glutamicum* CgHis2 starting strain. **Data S1.** Computational analysis of FAAMS data. **Data S2.** Rationale for selection of three SNPs for reconstruction in *C. glutamicum* CgHis2.

## Data Availability

Additional tables and figures underlying the article are available in the supplementary material (Additional file 1). The genome sequencing raw data of the 100 biosensor/FACS-isolated *C. glutamicum* variants are stored at Sequence Read Archive (SRA) of the National Library of Medicine (NCBI) and can be found under the BioProject ID PRJNA990379 [34].

## Abbreviations

AICAR: 5-Aminoimidazole-4-carboxamide ribonucleotide
BHI: Brain-heart-infusion medium
CGXII: Defined mineral medium for cultivation of *C. glutamicum*
EYFP: Enhanced yellow fluorescent protein
FAAMS: Fast Automated Analysis of Multiple Sequences
FACS: Fluorescence-activated cell sorting
FSC: Front scatter
HisE: Phosphoribosyl-ATP pyrophosphatase
HisG: ATP phosphoribosyltransferase
InDel: Insertion/deletion
LTTR: LysR-type transcriptional regulator
MNNG: *N*-Methyl-*N'*-nitro-*N*-nitrosoguanidine
NRPS: Non-ribosomal peptide synthetase
SNP: Single-nucleotide polymorphism
SSC: Side scatter
WGS: Whole genome sequencing
