# Supplementary material for: Beyond rational—biosensor-guided isolation of 100 independently evolved bacterial strain variants and comparative analysis of their genomes
Source: BMC Biol. 2023 Sep 4;21:183. doi: 10.1186/s12915-023-01688-x (PMC10478468; doi:10.1186/s12915-023-01688-x)
Supplement: Supplementary file 1 — Additional file 1: Figure S1. Comparison of C. glutamicum CgHis1 pHisOP1 and its variant C. glutamicum CgHis2, which served as screening host. Figure S2. Specific fluorescence response of biosensor-harboring C. glutamicum strains upon initial dipeptide supplementation. Figure S3. Biosensor crosstalk – Co-cultivation of C. glutamicum CgHis2 and C. glutamicum wild type pSenHis. Figure S4. Schematic overview of the screening workflow for the identification of significantly improved l-histidine producing C. glutamicum CgHis2 strain variants. Figure S5. Individual SNP-distribution across the genome of all 100 FACS-isolated and l -histidine producing C. glutamicum CgHis2 variants. Figure S6. Mutational bias of MNNG-mutagenesis. Figure S7. Predicted structure of NCgl2981 and position of the D735 residue as calculated by AlphaFold. Figure S8. l-histidine production performance of reverse engineered CgHis2 single mutant- and double mutant strains. Figure S9. l-histidine production performance of reverse engineered CgHis2 variants in comparison to the CgHis2 reference strain. Figure S10. Genomic position of the cps-gene encoding the non-ribosomal peptide synthase with an unknown MarR-type regulator gene located directly upstream in C. glutamicum ATCC 13032. The position of the cps-gene and the gene of the MarR-type regulator gene are highly conserved among various Corynebacterium species. Figure S11.Genomic position of the NCgl2981 gene of unknown function in C. glutamicum ATCC 13032. NCgl2981 is highly conserved among various Corynebacterium and Mycobacterium species. Table S1. Bacterial strains and plasmids. Table S2. Oligonucleotides. Table S3. Identified mutations in already known genetic targets contributing to l-histidine production from 100 improved C. glutamicum CgHis2 variants. Table S4. Hotspot genes in C. glutamicum CgHis2 identified by computational analysis of the FAAMS dataset. Table S5. List of SNPs identified in 100 independently isolated l-histidine producing [file 12915_2023_1688_MOESM1_ESM.doc]

**Beyond rational - biosensor-guided isolation of 100 independently evolved bacterial strain variants and comparative analysis of their genomes**

**Philipp T. Baumann^1^, Michael Dal Molin^1,2,3^, Hannah Aring^1^, Karin Krumbach^1^, Moritz-Fabian Müller^1^, Bas Vroling^4^, Philana V. van Summeren-Wesenhagen^5^, Stephan Noack^1^, Jan Marienhagen^1,6 *^**

**^1^**Institute of Bio- and Geosciences, IBG-1: Biotechnology, Forschungszentrum Jülich, D-52425 Jülich, Germany

^2^Department I of Internal Medicine, University of Cologne, D-50937 Cologne, Germany.

^3^Center for Molecular Medicine Cologne (CMMC), University of Cologne, D-50931 Cologne, Germany

^4^Bioprodict GmbH, Nieuwe Marktstraat 54E, 6511AA Nijmegen, The Netherlands

^5^SenseUp GmbH, c/o Campus Forschungszentrum, Wilhelm-Johnen-Strasse, D-52428, Jülich, Germany

^6^Institute of Biotechnology, RWTH Aachen University, Worringer Weg 3, D-52074 Aachen, Germany

^*^ Corresponding author:

Prof. Dr. Jan Marienhagen, phone: +49 2461 61 2843, e-mail: j.marienhagen@fz-juelich.de
ORCID ID: 0000-0001-5513-3730

## *C. glutamicum* CgHis1 – engineered for l‑histidine production

**Table S1:** **Bacterial strains and plasmids.**

| **Strain or plasmid** | **Relevant characteristics** | **Source/Reference** |
| --- | --- | --- |
| ***E. coli* strains** | | |
| **DH5α** | F– Φ80*lacZ*ΔM15 Δ(*lacZYA-argF*)U169  *recA1 endA1 hsdR17* (rK–, mK+) *phoA*  *supE44* λ– *thi*-1 *gyrA96* *relA1* | Invitrogen (Karlsruhe, Germany) |
|  |  |  |
| ***C. glutamicum* strains** | | |
| **wild type** | wild type strain, ATCC 13032, biotin-auxotroph |  |
| ***ΔlysEG*** | wild type with deletion of *lysE* (encoding basic amino acid transporter LysE) and *lysG* (encoding transcriptional regulator of *lysE*) | [1] |
| **CgHis1** | engineered l‑histidine producer strain,  *C. glutamicum ATCC13032*  derivative with P_trc_-*hisEG(S143F/ΔC)*, P_trc_-*hisDCB*, P*tuf*-*hisHAFI*, P_H36_-*hisN*, P_tuf_-*fbp*, ∆*iolR* | Senseup GmbH (Jülich, Germany) |
| **CgHis2** | engineered l‑histidine producer strain, based on CgHis1 with deletion of *lysEG* and harboring the pSenHis[*hisEG*]-biosensor plasmid | this study |
| **CgHis2 derivatives** | **Isolates from FACS-screening** | |
| **strain nomenclature X1-X2-X3-X4 according to X1, # mutagenesis; X2, MNNG condition (i.e. 50 = 5.0 mg mL^‑1^); X3, column number in mutagenesis plate; X4, clone number after single cell sorting** | | |
| **1-50-81-2** | mutagenized CgHis2 derivative, isolated in pSenHis-based FACS-screening | this study |
| **1-50-44-2** | mutagenized CgHis2 derivative, isolated in pSenHis-based FACS-screening | this study |
| **1-50-39-12** | mutagenized CgHis2 derivative, isolated in pSenHis-based FACS-screening | this study |
| **1-50-38-2** | mutagenized CgHis2 derivative, isolated in pSenHis-based FACS-screening | this study |
| **1-50-1-8** | mutagenized CgHis2 derivative, isolated in pSenHis-based FACS-screening | this study |
| **1-50-15-6** | mutagenized CgHis2 derivative, isolated in pSenHis-based FACS-screening | this study |
| **6-50-398-14** | mutagenized CgHis2 derivative, isolated in pSenHis-based FACS-screening | this study |
| **7-60-9-11** | mutagenized CgHis2 derivative, isolated in pSenHis-based FACS-screening | this study |
| **7-60-6-4** | mutagenized CgHis2 derivative, isolated in pSenHis-based FACS-screening | this study |
| **7-60-5-9** | mutagenized CgHis2 derivative, isolated in pSenHis-based FACS-screening | this study |
| **7-60-12-1** | mutagenized CgHis2 derivative, isolated in pSenHis-based FACS-screening | this study |
| **7-60-11-2** | mutagenized CgHis2 derivative, isolated in pSenHis-based FACS-screening | this study |
| **7-60-10-21** | mutagenized CgHis2 derivative, isolated in pSenHis-based FACS-screening | this study |
| **7-55-8-5** | mutagenized CgHis2 derivative, isolated in pSenHis-based FACS-screening | this study |
| **7-55-11-16** | mutagenized CgHis2 derivative, isolated in pSenHis-based FACS-screening | this study |
| **7-55-9-22** | mutagenized CgHis2 derivative, isolated in pSenHis-based FACS-screening | this study |
| **7-55-5-16** | mutagenized CgHis2 derivative, isolated in pSenHis-based FACS-screening | this study |
| **7-55-4-6** | mutagenized CgHis2 derivative, isolated in pSenHis-based FACS-screening | this study |
| **7-55-10-21** | mutagenized CgHis2 derivative, isolated in pSenHis-based FACS-screening | this study |
| **7-50-8-15** | mutagenized CgHis2 derivative, isolated in pSenHis-based FACS-screening | this study |
| **7-50-6-18** | mutagenized CgHis2 derivative, isolated in pSenHis-based FACS-screening | this study |
| **7-50-4-15** | mutagenized CgHis2 derivative, isolated in pSenHis-based FACS-screening | this study |
| **7-50-11-21** | mutagenized CgHis2 derivative, isolated in pSenHis-based FACS-screening | this study |
| **7-50-10-8** | mutagenized CgHis2 derivative, isolated in pSenHis-based FACS-screening | this study |
| **7-45-7-14** | mutagenized CgHis2 derivative, isolated in pSenHis-based FACS-screening | this study |
| **7-45-6-2** | mutagenized CgHis2 derivative, isolated in pSenHis-based FACS-screening | this study |
| **7-45-5-5** | mutagenized CgHis2 derivative, isolated in pSenHis-based FACS-screening | this study |
| **7-45-4-16** | mutagenized CgHis2 derivative, isolated in pSenHis-based FACS-screening | this study |
| **7-45-11-24** | mutagenized CgHis2 derivative, isolated in pSenHis-based FACS-screening | this study |
| **7-45-10-9** | mutagenized CgHis2 derivative, isolated in pSenHis-based FACS-screening | this study |
| **7-40-9-14** | mutagenized CgHis2 derivative, isolated in pSenHis-based FACS-screening | this study |
| **7-40-8-23** | mutagenized CgHis2 derivative, isolated in pSenHis-based FACS-screening | this study |
| **7-40-7-8** | mutagenized CgHis2 derivative, isolated in pSenHis-based FACS-screening | this study |
| **7-40-6-4** | mutagenized CgHis2 derivative, isolated in pSenHis-based FACS-screening | this study |
| **7-40-5-24** | mutagenized CgHis2 derivative, isolated in pSenHis-based FACS-screening | this study |
| **7-40-4-11** | mutagenized CgHis2 derivative, isolated in pSenHis-based FACS-screening | this study |
| **7-40-12-7** | mutagenized CgHis2 derivative, isolated in pSenHis-based FACS-screening | this study |
| **7-40-11-5** | mutagenized CgHis2 derivative, isolated in pSenHis-based FACS-screening | this study |
| **7-40-10-2** | mutagenized CgHis2 derivative, isolated in pSenHis-based FACS-screening | this study |
| **7-35-9-3** | mutagenized CgHis2 derivative, isolated in pSenHis-based FACS-screening | this study |
| **7-35-7-14** | mutagenized CgHis2 derivative, isolated in pSenHis-based FACS-screening | this study |
| **7-35-6-8** | mutagenized CgHis2 derivative, isolated in pSenHis-based FACS-screening | this study |
| **7-35-5-20** | mutagenized CgHis2 derivative, isolated in pSenHis-based FACS-screening | this study |
| **7-35-4-1** | mutagenized CgHis2 derivative, isolated in pSenHis-based FACS-screening | this study |
| **7-35-12-20** | mutagenized CgHis2 derivative, isolated in pSenHis-based FACS-screening | this study |
| **7-35-11-20** | mutagenized CgHis2 derivative, isolated in pSenHis-based FACS-screening | this study |
| **7-30-9-20** | mutagenized CgHis2 derivative, isolated in pSenHis-based FACS-screening | this study |
| **7-30-8-11** | mutagenized CgHis2 derivative, isolated in pSenHis-based FACS-screening | this study |
| **7-30-7-3** | mutagenized CgHis2 derivative, isolated in pSenHis-based FACS-screening | this study |
| **7-30-6-15** | mutagenized CgHis2 derivative, isolated in pSenHis-based FACS-screening | this study |
| **7-30-5-7** | mutagenized CgHis2 derivative, isolated in pSenHis-based FACS-screening | this study |
| **7-30-10-1** | mutagenized CgHis2 derivative, isolated in pSenHis-based FACS-screening | this study |
| **7-25-9-18** | mutagenized CgHis2 derivative, isolated in pSenHis-based FACS-screening | this study |
| **7-25-6-10** | mutagenized CgHis2 derivative, isolated in pSenHis-based FACS-screening | this study |
| **7-25-5-21** | mutagenized CgHis2 derivative, isolated in pSenHis-based FACS-screening | this study |
| **7-25-4-3** | mutagenized CgHis2 derivative, isolated in pSenHis-based FACS-screening | this study |
| **10-30-9-12** | mutagenized CgHis2 derivative, isolated in pSenHis-based FACS-screening | this study |
| **10-30-12-9** | mutagenized CgHis2 derivative, isolated in pSenHis-based FACS-screening | this study |
| **10-30-11-11** | mutagenized CgHis2 derivative, isolated in pSenHis-based FACS-screening | this study |
| **10-20-7-24** | mutagenized CgHis2 derivative, isolated in pSenHis-based FACS-screening | this study |
| **10-20-5-16** | mutagenized CgHis2 derivative, isolated in pSenHis-based FACS-screening | this study |
| **10-20-4-20** | mutagenized CgHis2 derivative, isolated in pSenHis-based FACS-screening | this study |
| **10-20-10-20** | mutagenized CgHis2 derivative, isolated in pSenHis-based FACS-screening | this study |
| **10-10-6-13** | mutagenized CgHis2 derivative, isolated in pSenHis-based FACS-screening | this study |
| **10-10-4-2** | mutagenized CgHis2 derivative, isolated in pSenHis-based FACS-screening | this study |
| **11-25-5-6** | mutagenized CgHis2 derivative, isolated in pSenHis-based FACS-screening | this study |
| **11-15-9-18** | mutagenized CgHis2 derivative, isolated in pSenHis-based FACS-screening | this study |
| **11-15-4-6** | mutagenized CgHis2 derivative, isolated in pSenHis-based FACS-screening | this study |
| **11-10-7-14** | mutagenized CgHis2 derivative, isolated in pSenHis-based FACS-screening | this study |
| **11-10-6-22** | mutagenized CgHis2 derivative, isolated in pSenHis-based FACS-screening | this study |
| **11-10-5-2** | mutagenized CgHis2 derivative, isolated in pSenHis-based FACS-screening | this study |
| **11-10-11-7** | mutagenized CgHis2 derivative, isolated in pSenHis-based FACS-screening | this study |
| **12-20-7-18** | mutagenized CgHis2 derivative, isolated in pSenHis-based FACS-screening | this study |
| **12-20-4-16** | mutagenized CgHis2 derivative, isolated in pSenHis-based FACS-screening | this study |
| **12-20-15-17** | mutagenized CgHis2 derivative, isolated in pSenHis-based FACS-screening | this study |
| **12-20-14-11** | mutagenized CgHis2 derivative, isolated in pSenHis-based FACS-screening | this study |
| **12-20-12-3** | mutagenized CgHis2 derivative, isolated in pSenHis-based FACS-screening | this study |
| **12-15-9-18** | mutagenized CgHis2 derivative, isolated in pSenHis-based FACS-screening | this study |
| **12-15-8-5** | mutagenized CgHis2 derivative, isolated in pSenHis-based FACS-screening | this study |
| **12-15-7-9** | mutagenized CgHis2 derivative, isolated in pSenHis-based FACS-screening | this study |
| **12-15-6-10** | mutagenized CgHis2 derivative, isolated in pSenHis-based FACS-screening | this study |
| **12-15-5-4** | mutagenized CgHis2 derivative, isolated in pSenHis-based FACS-screening | this study |
| **12-15-4-15** | mutagenized CgHis2 derivative, isolated in pSenHis-based FACS-screening | this study |
| **12-15-21-16** | mutagenized CgHis2 derivative, isolated in pSenHis-based FACS-screening | this study |
| **12-15-20-1** | mutagenized CgHis2 derivative, isolated in pSenHis-based FACS-screening | this study |
| **12-15-19-24** | mutagenized CgHis2 derivative, isolated in pSenHis-based FACS-screening | this study |
| **12-15-18-8** | mutagenized CgHis2 derivative, isolated in pSenHis-based FACS-screening | this study |
| **12-15-16-9** | mutagenized CgHis2 derivative, isolated in pSenHis-based FACS-screening | this study |
| **12-15-15-6** | mutagenized CgHis2 derivative, isolated in pSenHis-based FACS-screening | this study |
| **12-15-12-5** | mutagenized CgHis2 derivative, isolated in pSenHis-based FACS-screening | this study |
| **12-15-10-7** | mutagenized CgHis2 derivative, isolated in pSenHis-based FACS-screening | this study |
| **12-10-9-5** | mutagenized CgHis2 derivative, isolated in pSenHis-based FACS-screening | this study |
| **12-10-8-6** | mutagenized CgHis2 derivative, isolated in pSenHis-based FACS-screening | this study |
| **12-10-7-2** | mutagenized CgHis2 derivative, isolated in pSenHis-based FACS-screening | this study |
| **12-10-6-23** | mutagenized CgHis2 derivative, isolated in pSenHis-based FACS-screening | this study |
| **12-10-5-6** | mutagenized CgHis2 derivative, isolated in pSenHis-based FACS-screening | this study |
| **12-10-4-1** | mutagenized CgHis2 derivative, isolated in pSenHis-based FACS-screening | this study |
| **12-10-12-5** | mutagenized CgHis2 derivative, isolated in pSenHis-based FACS-screening | this study |
| **12-10-11-11** | mutagenized CgHis2 derivative, isolated in pSenHis-based FACS-screening | this study |
| **12-10-10-16** | mutagenized CgHis2 derivative, isolated in pSenHis-based FACS-screening | this study |
| **CgHis2 derivatives** | **reverse engineered CgHis2 strains harboring point mutations in identified hotspot genes** | |
| ***cps*-G987D** | CgHis2 derivative harboring point mutation in *cps* leading to amino acid substitution G987D | this study |
| ***emb* -G477E** | CgHis2 derivative harboring point mutation in *emb* leading to amino acid substitution G477E | this study |
| ***emb*-T529I/T539I** | CgHis2 derivative harboring point mutation in *emb* leading to amino acid substitutions T529I/T539I | this study |
| ***fasA*-A2702T** | CgHis2 derivative harboring point mutation in *fasA* leading to amino acid substitution A2702T | this study |
| ***fasA*-P783S** | CgHis2 derivative harboring point mutation in *fasA* leading to amino acid substitution P783S | this study |
| ***fasB*-G1921E** | CgHis2 derivative harboring point mutation in *fasB* leading to amino acid substitution G1921E | this study |
| ***fasB*-G2762D** | CgHis2 derivative harboring point mutation in *fasB* leading to amino acid substitution G2762D | this study |
| ***gltB*-G1106D** | CgHis2 derivative harboring point mutation in *gltB* leading to amino acid substitution G1106D | this study |
| ***gltB*-P988S** | CgHis2 derivative harboring point mutation in *gltB* leading to amino acid substitution P988S | this study |
| ***iolD*-S481F** | CgHis2 derivative harboring point mutation in *iolD* leading to amino acid substitution S481F | this study |
| ***mrpA*-L42F** | CgHis2 derivative harboring point mutation in *mrpA* leading to amino acid substitution L42F | this study |
| ***NCgl0552*-G432D** | CgHis2 derivative harboring point mutation in *NCgl0552* leading to amino acid substitution G432D | this study |
| ***NCgl0552*-P823S** | CgHis2 derivative harboring point mutation in *NCgl0552* leading to amino acid substitution P823S | this study |
| ***NCgl0705*-S1847N** | CgHis2 derivative harboring point mutation in *NCgl0705* leading to amino acid substitution S1847N | this study |
| ***NCgl2789*-S265N** | CgHis2 derivative harboring point mutation in *NCgl2789* leading to amino acid substitution S265N | this study |
| ***NCgl2859*-S372F** | CgHis2 derivative harboring point mutation in *NCgl2859* leading to amino acid substitution S372F | this study |
| ***NCgl2959*-D1453N** | CgHis2 derivative harboring point mutation in *NCgl2959* leading to amino acid substitution D1453N | this study |
| ***NCgl2959*-G870D** | CgHis2 derivative harboring point mutation in *NCgl2959* leading to amino acid substitution G870D | this study |
| ***NCgl2964*-E512K** | CgHis2 derivative harboring point mutation in *NCgl2964* leading to amino acid substitution E512K | this study |
| ***NCgl2964*-P863S** | CgHis2 derivative harboring point mutation in *NCgl2964* leading to amino acid substitution P863S | this study |
| ***NCgl2981*-D735G** | CgHis2 derivative harboring point mutation in *NCgl2981* leading to amino acid substitution D735G | this study |
| ***pks*-A1525V** | CgHis2 derivative harboring point mutation in *pks* leading to amino acid substitution A1525V | this study |
| ***pks*-D1186N** | CgHis2 derivative harboring point mutation in *pks* leading to amino acid substitution D1186N | this study |
| ***putA*-P217S** | CgHis2 derivative harboring point mutation in *putA* leading to amino acid substitution P217S | this study |
| ***pyc*-A764V** | CgHis2 derivative harboring point mutation in *pyc* leading to amino acid substitution A764V | this study |
| ***pyk*-T357I** | CgHis2 derivative harboring point mutation in *pyk* leading to amino acid substitution T357I | this study |
| ***ulaA*-V219I** | CgHis2 derivative harboring point mutation in *ulaA* leading to amino acid substitution V219I | this study |
| ***xylB*-G55R** | CgHis2 derivative harboring point mutation in *xylB* leading to amino acid substitution G55R | this study |
| **CgHis2 derivatives** | **CgHis2 strain variants harboring in-frame deletions in specific hotspot genes** | |
| **Δ*cps*** | CgHis2 derivative harboring respective in-frame deletion | this study |
| **Δ*emb*** | CgHis2 derivative harboring respective in-frame deletion | this study |
| **Δ*fasB*** | CgHis2 derivative harboring respective in-frame deletion | this study |
| **Δ*ggtB*** | CgHis2 derivative harboring respective in-frame deletion | this study |
| **Δ*gltB*** | CgHis2 derivative harboring respective in-frame deletion | this study |
| **Δ*NCgl0552*** | CgHis2 derivative harboring respective in-frame deletion | this study |
| **Δ*NCgl0705*** | CgHis2 derivative harboring respective in-frame deletion | this study |
| **Δ*NCgl1737*** | CgHis2 derivative harboring respective in-frame deletion | this study |
| **Δ*NCgl2959*** | CgHis2 derivative harboring respective in-frame deletion | this study |
| **Δ*NCgl2964*** | CgHis2 derivative harboring respective in-frame deletion | this study |
| **Δ*putA*** | CgHis2 derivative harboring respective in-frame deletion | this study |
| **Δ*pyk1*** | CgHis2 derivative harboring respective in-frame deletion | this study |
| **Δ*pyk2*** | CgHis2 derivative harboring respective in-frame deletion | this study |
| ***NCgl0705*-S1847N *cps*-G987D** | CgHis2 derivative harboring *NCgl0705*-S1847N *cps*-G987D | this study |
| ***NCgl2981*-D735G *mrpA*-L42F** | CgHis2 derivative harboring *NCgl2981*-D735G *mrpA*-L42F | this study |
| **Δ*cps NCgl2981*-D735G** | CgHis2 derivative harboring Δ*cps NCgl2981*-D735G | this study |
| **Δ*cps pks*-D1186N** | CgHis2 derivative harboring Δ*cps pks*-D1186N | this study |
| **Δ*cps* Δ*pyk1*** | CgHis2 derivative harboring Δ*cps* Δ*pyk1* | this study |
| **Δ*cps* Δ*pyk1 NCgl2981*-D735G** | CgHis2 derivative harboring Δ*cps* Δ*pyk1 NCgl2981*-D735G | this study |
| **Δ*fasB NCgl2981*-D735G** | CgHis2 derivative harboring Δ*fasB NCgl2981*-D735G | this study |
| **Δ*fasB pks*-D1186N** | CgHis2 derivative harboring Δ*fasB pks*-D1186N | this study |
| **Δ*fasB* Δ*cps*** | CgHis2 derivative harboring Δ*fasB* Δ*cps* | this study |
| **Δ*fasB* Δ*cps NCgl2981*-D735G** | CgHis2 derivative harboring Δ*fasB* Δ*cps NCgl2981*-D735G | this study |
| **Δ*fasB* Δ*cps pks*-D1186N** | CgHis2 derivative harboring Δ*fasB* Δ*cps pks*-D1186N | this study |
| **Δ*fasB* Δ*cps* Δ*pyk1*** | CgHis2 derivative harboring Δ*fasB* Δ*cps* Δ*pyk1* | this study |
| **Δ*fasB* Δ*cps* Δ*pyk1 NCgl2981*-D735G** | CgHis2 derivative harboring Δ*fasB* Δ*cps* Δ*pyk1 NCgl2981*-D735G | this study |
| **Δ*fasB* Δ*cps* Δ*pyk1 pks*-D1186N** | CgHis2 derivative harboring Δ*fasB* Δ*cps* Δ*pyk1 pks*-D1186N | this study |
| **Δ*fasB* Δ*cps* Δ*pyk1 pks*-D1186N *NCgl2981*-D735G** | CgHis2 derivative harboring Δ*fasB* Δ*cps* Δ*pyk1 pks*-D1186N *NCgl2981*-D735G | this study |
| **Δ*fasB* Δ*pyk1*** | CgHis2 derivative harboring Δ*fasB* Δ*pyk1* | this study |
| **Δ*fasB* Δ*pyk1 NCgl2981*-D735G** | CgHis2 derivative harboring Δ*fasB* Δ*pyk1 NCgl2981*-D735G | this study |
| **Δ*fasB* Δ*pyk1 NCgl2981*-D735G *pks*-D1186N** | CgHis2 derivative harboring Δ*fasB* Δ*pyk1 NCgl2981*-D735G *pks*-D1186N | this study |
| **Δ*fasB* Δ*pyk1 pks*-D1186N** | CgHis2 derivative harboring Δ*fasB* Δ*pyk1 pks*-D1186N | this study |
| **Δ*pyk1 NCgl2981*-D735G** | CgHis2 derivative harboring Δ*pyk1 NCgl2981*-D735G | this study |
| **Δ*pyk1 pks*-D1186N** | CgHis2 derivative harboring Δ*pyk1 pks*-D1186N | this study |
| **Δ*pyk1*Δ*pyk2*** | CgHis2 derivative harboring Δ*pyk1*Δ*pyk2* | this study |
|  |  |  |
| **Plasmids** | | |
| **pk19*mobsacB*** | mobilizable suicide vector for double homologous recombination in *C. glutamicum*, kanamycin resistance, levansucrase gene *sacB* | [2] |
| **pSenHis** | pJC1-based biosensor plasmid, kanamycin resistance, LysG-A219L based biosensor module, *eYFP* as fluorescence reporter gene | [3] |
| **pSenHis[*hisEG*]** | pSenHis biosensor module subcloned onto pHisOP1, kanamycin resistance | this study |
| **pHisOP1** | pJC1-based plasmid for overexpression of *hisEG* operon with *hisG(S143F/ΔC)* variant | SenseUp GmbH |
| **pk19*mobsacB* derivatives** | | |
| **pk19-*cps*-G987D** | pk19*mobsacB* derivative for introduction of *cps*-G987D | this study |
| **pk19-*emb*-G477E** | pk19*mobsacB* derivative for introduction of *emb* -G477E | this study |
| **pk19-*emb*-T529I/T539I** | pk19*mobsacB* derivative for introduction of *emb*-T529I/T539I | this study |
| **pk19-*fasA*-A2702T** | pk19*mobsacB* derivative for introduction of *fasA*-A2702T | this study |
| **pk19-*fasA*-P783S** | pk19*mobsacB* derivative for introduction of *fasA*-P783S | this study |
| **pk19-*fasB*-G1921E** | pk19*mobsacB* derivative for introduction of *fasB*-G1921E | this study |
| **pk19-*fasB*-G2762D** | pk19*mobsacB* derivative for introduction of *fasB*-G2762D | this study |
| **pk19-*gltB*-G1106D** | pk19*mobsacB* derivative for introduction of *gltB*-G1106D | this study |
| **pk19-*gltB*-P988S** | pk19*mobsacB* derivative for introduction of *gltB*-P988S | this study |
| **pk19-*iolD*-S481F** | pk19*mobsacB* derivative for introduction of *iolD*-S481F | this study |
| **pk19-*mrpA*-L42F** | pk19*mobsacB* derivative for introduction of *mrpA*-L42F | this study |
| **pk19-*NCgl0552*-G432D** | pk19*mobsacB* derivative for introduction of *NCgl0552*-G432D | this study |
| **pk19-*NCgl0552*-P823S** | pk19*mobsacB* derivative for introduction of *NCgl0552*-P823S | this study |
| **pk19-*NCgl0705*-S1847N** | pk19*mobsacB* derivative for introduction of *NCgl0705*-S1847N | this study |
| **pk19-*NCgl2789*-S265N** | pk19*mobsacB* derivative for introduction of *NCgl2789*-S265N | this study |
| **pk19-*NCgl2859*-S372F** | pk19*mobsacB* derivative for introduction of *NCgl2859*-S372F | this study |
| **pk19-*NCgl2959*-D1453N** | pk19*mobsacB* derivative for introduction of *NCgl2959*-D1453N | this study |
| **pk19-*NCgl2959*-G870D** | pk19*mobsacB* derivative for introduction of *NCgl2959*-G870D | this study |
| **pk19-*NCgl2964*-E512K** | pk19*mobsacB* derivative for introduction of *NCgl2964*-E512K | this study |
| **pk19-*NCgl2964*-P863S** | pk19*mobsacB* derivative for introduction of *NCgl2964*-P863S | this study |
| **pk19-*NCgl2981*-D735G** | pk19*mobsacB* derivative for introduction of *NCgl2981*-D735G | this study |
| **pk19-*pks*-A1525V** | pk19*mobsacB* derivative for introduction of *pks*-A1525V | this study |
| **pk19-*pks*-D1186N** | pk19*mobsacB* derivative for introduction of *pks*-D1186N | this study |
| **pk19-*putA*-P217S** | pk19*mobsacB* derivative for introduction of *putA*-P217S | this study |
| **pk19-*pyc*-A764V** | pk19*mobsacB* derivative for introduction of *pyc*-A764V | this study |
| **pk19-*pyk*-T357I** | pk19*mobsacB* derivative for introduction of *pyk*-T357I | this study |
| **pk19-*ulaA*-V219I** | pk19*mobsacB* derivative for introduction of *ulaA*-V219I | this study |
| **pk19-*xylB*-G55R** | pk19*mobsacB* derivative for introduction of *xylB*-G55R | this study |
| **pk19-Δ*lysEG*** | pk19*mobsacB* derivative for in-frame deletion of *lysEG* | [1] |
| **pk19-Δ*cps*** | pk19*mobsacB* derivative for in-frame deletion of *cps* | this study |
| **pk19-Δ*emb*** | pk19*mobsacB* derivative for in-frame deletion of *emb* | this study |
| **pk19-Δ*fasB*** | pk19*mobsacB* derivative for in-frame deletion of *fasB* | [4] |
| **pk19-Δ*ggtB*** | pk19*mobsacB* derivative for in-frame deletion of *ggtB* | this study |
| **pk19-Δ*gltB*** | pk19*mobsacB* derivative for in-frame deletion of *gltB* | this study |
| **pk19-Δ*NCgl0552*** | pk19*mobsacB* derivative for in-frame deletion of *NCgl0552* | this study |
| **pk19-Δ*NCgl0705*** | pk19*mobsacB* derivative for in-frame deletion of *NCgl0705* | this study |
| **pk19-Δ*NCgl1737*** | pk19*mobsacB* derivative for in-frame deletion of *NCgl1737* | this study |
| **pk19-Δ*NCgl2959*** | pk19*mobsacB* derivative for in-frame deletion of *NCgl2959* | this study |
| **pk19-Δ*NCgl2964*** | pk19*mobsacB* derivative for in-frame deletion of *NCgl2964* | this study |
| **pk19-Δ*NCgl2981*** | pk19*mobsacB* derivative for in-frame deletion of *NCgl2981* | this study |
| **pk19-Δ*pknB*** | pk19*mobsacB* derivative for in-frame deletion of *pknB* | this study |
| **pk19-Δ*pks*** | pk19*mobsacB* derivative for in-frame deletion of *pks* | this study |
| **pk19-Δ*putA*** | pk19*mobsacB* derivative for in-frame deletion of *putA* | this study |
| **pk19-Δ*pyk1*** | pk19*mobsacB* derivative for in-frame deletion of *pyk1* | this study |
| **pk19-Δ*pyk2*** | pk19*mobsacB* derivative for in-frame deletion of *pyk2* | this study |

**Table S2: Oligonucleotides.**

| **Name** | **Type** | **Sequence** |
| --- | --- | --- |
| **Rsp_pK19 Check Primer1** | Sequencing Primer | CACAGGAAACAGCTATGACCATG |
| **Univ_pK19 Check Primer 2** | Sequencing Primer | CGCCAGGGTTTTCCCAGTCACGAC |
| **1_FasA 893775 G-A (A-T) up fragment_fwd** | Gibson Primer | ATCCCCGGGTACCGAGCTCGTCGACGCCTTCCTGTCCTC |
| **1_Fas A 893775 G-A (A-T) up fragment_rev** | Gibson Primer | GATGCACGTGTCAGGAGGACGGTACCGCC |
| **1_FasA 893775 G-A (A-T) down_fwd** | Gibson Primer | GTCCTCCTGACACGTGCATCCGTTGCTG |
| **1_FasA 893775 G-A (A-T) down_rev** | Gibson Primer | TTGTAAAACGACGGCCAGTGAATGGAGCCTGCGCCGAG |
| **1_FasA 893775 Check fwd** | Sequencing Primer | GACTTCGACCCTGCCAAGTG |
| **1_FasA 893775 Check rev** | Sequencing Primer | TGCGCATTGCCTGCTCGAAG |
| **2_gltB 198201 up fragment_fwd** | Gibson Primer | ATCCCCGGGTACCGAGCTCGGTTCTGGCAGCTACGAGATTTTC |
| **2_gltB 198201 up fragment_rev** | Gibson Primer | GTGGTGGAGAGGAAATCAGACCAACGCC |
| **2_gltB 198201 down fragment_fwd** | Gibson Primer | TCTGATTTCCTCTCCACCACACCACGATATTTAC |
| **2_gltB 198201 down fragment_rev** | Gibson Primer | TTGTAAAACGACGGCCAGTGGTGTTCAGCCTTGCCGGTG |
| **2_gltB 198201 Check fwd** | Sequencing Primer | ATACAAGTGGCGCCGCGAAG |
| **2_gltB 198201 Check rev** | Sequencing Primer | GCACCTGGGCTTGTCCGACG |
| **3_pyk 3111252 up fragment_fwd** | Gibson Primer | ATCCCCGGGTACCGAGCTCGAGCCGAGTTCGACGGCGA |
| **3_pyk 3111252 up fragment_rev** | Gibson Primer | CCGGCCGCATACCACGCATCAGCTGCAC |
| **3_pyk 3111252 down fragment_fwd** | Gibson Primer | GATGCGTGGTATGCGGCCGGATCCGAGGG |
| **3_pyk 3111252 down fragment _rev** | Gibson Primer | TTGTAAAACGACGGCCAGTGCCGGGAAATCCGCGTCAGC |
| **3_pyk 3111252 Check fwd** | Sequencing Primer | GGACTTGGGTGGCCAAGATG |
| **3_pyk 3111252 Check rev** | Sequencing Primer | AAGCGGCATGAATCTAGCTC |
| **4_iolD_174806_up_fwd** | Gibson Primer | CCTGCAGGTCGACTCTAGAGGATCCCACCACCGCATCTCGCAC |
| **4_iolD_174806_up_rev** | Gibson Primer | CATGAGGTAGAACCCATCACCAACCATGATCAC |
| **4_iolD_174806_down_fwd** | Gibson Primer | GTGATGGGTTCTACCTCATGCTCAACAC |
| **4_iolD_174806_down_rev** | Gibson Primer | TTGTAAAACGACGGCCAGTGAATTCTAAATACGTTTTGGTTTAGCC |
| **4_iolD_Check_fwd** | Sequencing Primer | TTGCGGGTGATGCTGATGTG |
| **4_iolD_Check_rev** | Sequencing Primer | CAGCCACAGATGAAGCTTTG |
| **5_ulaA_up_fwd** | Gibson Primer | CCTGCAGGTCGACTCTAGAGGATCCACCCGTGGCATTACCGTAAAC |
| **5_ulaA_up_rev** | Gibson Primer | GATATGAAGATTCCGGAAGGCTTGCGCT |
| **5_ulaA_down_fwd** | Gibson Primer | CCTTCCGGAATCTTCATATCCTCAGTGGAAGGG |
| **5_ulaA_down_rev** | Gibson Primer | TTGTAAAACGACGGCCAGTGAATTCTGCTCATTGGTGCGGGTG |
| **5_ulaA_Check_fwd** | Sequencing Primer | CAGAAGACCGTTGGCAAAGG |
| **5_ulaA_Check_rev** | Sequencing Primer | ATCGGTGGAGCAATCAAAGC |
| **6_fasB_up_fwd** | Gibson Primer | CCTGCAGGTCGACTCTAGAGGATCCACAAGATCTGCCTTTGGTGCCTTCG |
| **6_fasB_up_rev** | Gibson Primer | CAGGCCTCGATGCCCTTGGTGCTGCTCG |
| **6_fasB_down_fwd** | Gibson Primer | ACCAAGGGCATCGAGGCCTGGGGCTGGGA |
| **6_fasB_down_rev** | Gibson Primer | TTGTAAAACGACGGCCAGTGAATTCCGGTGGCTACGGACAGATGATCC |
| **6_fasB_Check_fwd** | Sequencing Primer | ATCGCAACCAGAGCGGAGAC |
| **6_fasB_Check_rev** | Sequencing Primer | CAACGACGTTCTGCAGGAAG |
| **7_pks_up_fwd** | Gibson Primer | CCTGCAGGTCGACTCTAGAGGATCCACACCGTCAGAGTAAGGC |
| **7_pks_up_rev** | Gibson Primer | GCATCCTGGTTAAACTTAATTTTGCAGACTGGG |
| **7_pks_down_fwd** | Gibson Primer | ATTAAGTTTAACCAGGATGCGGTTGTCCAC |
| **7_pks_down_rev** | Gibson Primer | TTGTAAAACGACGGCCAGTGAATTCTTCTTCCGTGGTCTACCAACC |
| **7_pks_Check_fwd** | Sequencing Primer | ATGGGCGTAGATGGCCACTG |
| **7_pks_Check_rev** | Sequencing Primer | AGCCTGCAGTGTTCATGTTC |
| **8_emb_up_fwd** | Gibson Primer | CCTGCAGGTCGACTCTAGAGGATCCTTTTCACATCGTGGACAAAG |
| **8_emb_up_rev** | Gibson Primer | TCTGGTACCACGAATATGTGCGCTACCAAATCGTCATGGAACAAACCGTTG |
| **8_emb_down_fwd** | Gibson Primer | ATTTGGTAGCGCACATATTCGTGGTACCAGATCAGTGCCGGGCCCTTCG |
| **8_emb_down_rev** | Gibson Primer | TTGTAAAACGACGGCCAGTGAATTCCGTGATCACCAGAGAGGTCATGC |
| **8_emb_Check_fwd** | Sequencing Primer | GTTCGCCCATGGAGTGATTG |
| **8_emb_Check_rev** | Sequencing Primer | TTCGTCTACCCGCATTGCTC |
| **9_NRPS_up_fwd** | Gibson Primer | CCTGCAGGTCGACTCTAGAGGATCCTGCAAAGCACCGCTTCCC |
| **9_NRPS_up_rev** | Gibson Primer | CTCATAGCCATCGATGAGGGTGTCATCGG |
| **9_NRPS_down_fwd** | Gibson Primer | CCCTCATCGATGGCTATGAGCTGGGTAATGG |
| **9_NRPS_down_rev** | Gibson Primer | TTGTAAAACGACGGCCAGTGAATTCAGCGGCTGAAGAGAGGGTG |
| **9_NRPS_Check_fwd** | Sequencing Primer | GACCATCAAGCCTGGTTCTC |
| **9_NRPS_Check_rev** | Sequencing Primer | GATTCCACGAACGCATCTTG |
| **10_DNSSeg_up_fwd** | Gibson Primer | CCTGCAGGTCGACTCTAGAGGATCCGTCGCCATCACCTCGCAC |
| **10_DNSSeg_up_rev** | Gibson Primer | ACTTTCTCTGAATCTTTGCGTCCTGCCAC |
| **10_DNSSeg_down_fwd** | Gibson Primer | CGCAAAGATTCAGAGAAAGTCCGCCGAG |
| **10_DNSSeg_down_rev** | Gibson Primer | TTGTAAAACGACGGCCAGTGAATTCCCGTGCAATCGGGATGCC |
| **10_DNSSeg_Check_fwd** | Sequencing Primer | CCGCTTCCAAGCAAGTTATG |
| **10_DNSSeg_Check_rev** | Sequencing Primer | TCCCACGTCAGCGTGGATAG |
| **13_helicase Check rev** | Gibson Primer | CTGAGCCCTTTGATCTTGTC |
| **13_helicase Check fwd** | Gibson Primer | ATCGCTTCTTGCCACTGTTC |
| **13_helicase up fwd** | Gibson Primer | CCTGCAGGTCGACTCTAGAGGATCCTTCAGCACTGTCATAGGG |
| **13_helicase up rev** | Gibson Primer | ACTGAGCTTAAGGTTAAAAACGATGACG |
| **13_helicase down fwd** | Sequencing Primer | TTTTTAACCTTAAGCTCAGTCGTATAAGCATTG |
| **13_helicase down rev** | Sequencing Primer | TTGTAAAACGACGGCCAGTGAATTCCCACAGGCATCACCTTGG |
| **14_hypo up fwd** | Gibson Primer | CCTGCAGGTCGACTCTAGAGGATCCAGACGGCACCTGGTCACAG |
| **14_hypo up rev** | Gibson Primer | GCAGCGCAGATTTTCGTCCGCACCCCAAC |
| **14 hypo_down fwd** | Gibson Primer | CGGACGAAAATCTGCGCTGCTCAAACCC |
| **14_hypo down rev** | Gibson Primer | TTGTAAAACGACGGCCAGTGAATTCCAGCATCGCCAGGACATG |
| **14_hypo Check fwd** | Sequencing Primer | TCCAGCGTGACCAGCAATTC |
| **14_hypo Check rev** | Sequencing Primer | TAGTCGTGCCACCTGTGTTG |
| **15_phosphatase up fwd** | Gibson Primer | CCTGCAGGTCGACTCTAGAGGATCCACTTTGATAACCAAGGCCAAAAG |
| **15_phosphatase up rev** | Gibson Primer | GTCACATAGTTCCAAATTCCGGGGACAAATG |
| **15_phosphatase down fwd** | Gibson Primer | GGAATTTGGAACTATGTGACCAACGCATTC |
| **15_phosphatase down rev** | Gibson Primer | TTGTAAAACGACGGCCAGTGAATTCGTGCTGACTGGAGTGGTG |
| **15_phosphatase Check fwd** | Sequencing Primer | TTCGATTACCAAGGCCTACC |
| **15_phosphatase Check rev** | Sequencing Primer | GCGTTTGCGCTTGGATCTTC |
| **16_emb up fwd** | Gibson Primer | CCTGCAGGTCGACTCTAGAGGATCCACGCACCGATGGAAATGG |
| **16_emb up rev** | Gibson Primer | GCGTCGAGGGAGGCGTCGAAAAGCAAAGTC |
| **16_emb down fwd** | Gibson Primer | TTCGACGCCTCCCTCGACGCCCCAATAAG |
| **16_emb down rev** | Gibson Primer | TTGTAAAACGACGGCCAGTGAATTCCGCGGATTATATGGCCAAC |
| **16_emb Check fwd** | Sequencing Primer | GCTTCGATGCCCTTGATCTG |
| **16_emb Check rev** | Sequencing Primer | CAAGCTGAAGCCACTTGATG |
| **17_DNA seg up fwd** | Gibson Primer | CCTGCAGGTCGACTCTAGAGGATCCGTGGCTGAAATGGGTACCG |
| **17_DNA seg up rev** | Gibson Primer | TCCCATTCCGTCGTGCGCCGATTCCTTCAG |
| **17_DNA seg down fwd** | Gibson Primer | CGGCGCACGACGGAATGGGACCACATGG |
| **17_DNA seg down rev** | Gibson Primer | TTGTAAAACGACGGCCAGTGAATTCGGGAATCCAATCCGCGCAAC |
| **17_DNA seg Check fwd** | Sequencing Primer | CGCTGGTTGTGCAGTTGTTG |
| **17_DNS seg Check rev** | Sequencing Primer | CCGGGTTGGCTGGGTAATTG |
| **18_fasB up fwd** | Gibson Primer | CCTGCAGGTCGACTCTAGAGGATCCGATCCTTGTAGAACTCCAGG |
| **18_fasB up rev** | Gibson Primer | AGGTCACCGAACACGATGGTGTGCTTGC |
| **18_fasB down fwd** | Gibson Primer | ACCATCGTGTTCGGTGACCTGCTCTGCAAAC |
| **18_fasB down rev** | Gibson Primer | TTGTAAAACGACGGCCAGTGAATTCCTCTCGCCGTAACCAGCTC |
| **18_fasB Check fwd** | Sequencing Primer | ATCCGACCCAGTTGATGATG |
| **18_fasB down rev** | Sequencing Primer | CAGCTTCACTCCTTCTGATG |
| **19_pyc up fwd** | Gibson Primer | CCTGCAGGTCGACTCTAGAGGATCCTTCGCGGCCGCAACACCG |
| **19_pyc up rev** | Gibson Primer | CCGTCAACAACATCTGCACCAGCTTGAGCTGC |
| **19_pyc down fwd** | Gibson Primer | GGTGCAGATGTTGTTGACGGTGCTTCCGC |
| **19_pyc down rev** | Gibson Primer | TTGTAAAACGACGGCCAGTGAATTCATGACAGAGTCTGGGATGTCG |
| **19_pyc Check fwd** | Sequencing Primer | ACGATGTGGCGATGCGTTTC |
| **19_pyc Check rev** | Sequencing Primer | GGCTATTGCGACGTTCCTTG |
| **20_pks_up fwd_new** | Gibson Primer | CCTGCAGGTCGACTCTAGAGGATCCAGTGGTTCGAGGGTTTCTG |
| **20_pks_up rev_new** | Gibson Primer | CATCGAAAATAACTTCCAGATCCCACCAC |
| **20_pks_down fwd_new** | Gibson Primer | TCTGGAAGTTATTTTCGATGCGGTTTTTGATGC |
| **20_pks_down rev_new** | Gibson Primer | TTGTAAAACGACGGCCAGTGAATTCCGACATGCTGCCACCAAG |
| **20_pks_Check_fwd** | Sequencing Primer | AGACGACGCATTAGTGGTTG |
| **20_pks_Check_rev** | Sequencing Primer | TCCGCGTGAATCTGCCAAAC |
| **21_fasA up fwd** | Gibson Primer | CCTGCAGGTCGACTCTAGAGGATCCCTGGCGGCTGGGTTGGCC |
| **21_fasA up rev** | Gibson Primer | GAACCCAAGACATAGGCTTGTGGTGCTTGCGG |
| **21_fasA down fwd** | Gibson Primer | CAAGCCTATGTCTTGGGTTCCAGCAATCG |
| **21_fasA down rev** | Gibson Primer | TTGTAAAACGACGGCCAGTGAATTCCTCACGGGAAACAACTGG |
| **21_fasA Check fwd** | Sequencing Primer | AAGCAGGCACTGGTCGACAC |
| **21_fasA Check rev** | Sequencing Primer | GCGGAAACAGATCCAACACC |
| **22_mrpA_up_fwd** | Gibson Primer | CCTGCAGGTCGACTCTAGAGGATCCGCAATAACATTCATTCCC |
| **22_mrpA_up_rev** | Gibson Primer | ACTCCGAAAACACCCAGAAAAAACCAATG |
| **22_mrpA_down_fwd** | Gibson Primer | TTTCTGGGTGTTTTCGGAGTTCATCAAAGGCAC |
| **22_mrpA_down_rev** | Gibson Primer | TTGTAAAACGACGGCCAGTGAATTCCCCAGGAGCTTGAGTAGG |
| **22_mrpA_Check_fwd** | Sequencing Primer | ACAACGTTTCCCGCACCTGG |
| **22_mrpA_Check_rev** | Sequencing Primer | CACATCTGCCCAGGAGCTTG |
| **23_helicase_up_fwd** | Gibson Primer | CCTGCAGGTCGACTCTAGAGGATCCAACCTTAGCCCACTTCATC |
| **23_helicase_up_rev** | Gibson Primer | AAATCTACTTTCAGATCTTGCTATGTGGG |
| **23_helicase_down_fwd** | Gibson Primer | CAAGATCTGAAAGTAGATTTGTTGCAAGG |
| **23_helicase_down_rev** | Gibson Primer | TTGTAAAACGACGGCCAGTGAATTCATAAGAAAAACAAATCTGCATTAAAG |
| **23_heicase_Check_fwd** | Sequencing Primer | GCGATGGCAGAACGTGAACC |
| **23_helicase_Check_rev** | Sequencing Primer | TGCCGTTGCCACAATTGAGG |
| **24_phosphatase_up_fwd** | Gibson Primer | CCTGCAGGTCGACTCTAGAGGATCCTTTCTTCGATGCCACCTTC |
| **24_phosphatase_up_rev** | Gibson Primer | ACAGTATCGTCACTTGGATCACTTGCTC |
| **24_phosphatase_down_fwd** | Gibson Primer | GATCCAAGTGACGATACTGTCAAGATGACGTTCCTGGAAG |
| **24_phosphatase_down_rev** | Gibson Primer | TTGTAAAACGACGGCCAGTGAATTCGGTGCGCTGCGGTCACGT |
| **24_phosphatase_Check fwd** | Sequencing Primer | CGACTAATTCTGCCTGATGG |
| **24_phosphatase_Check rev** | Sequencing Primer | GCACGTAGTCATGGATGTTG |
| **25_gltB_up_fwd** | Gibson Primer | CCTGCAGGTCGACTCTAGAGGATCCCCACTACTTGAACAACTGC |
| **25_gltB_up_rev** | Gibson Primer | TTCTTCGGCATCGAGAAGAGCTGCGATAAC |
| **25_gltB_down_fwd** | Gibson Primer | CTCTTCTCGATGCCGAAGAATTCGGTTTTG |
| **25_gltB_down_rev** | Gibson Primer | TTGTAAAACGACGGCCAGTGAATTCCAACTGAACGGTTGACGTTG |
| **25_gltB_check_fwd** | Sequencing Primer | CTGGGCGGTATGTCCAACTC |
| **25_gltB_check_rev** | Sequencing Primer | TGGGATGAACGCGCCAAAGG |
| **26_hypo_up_fwd** | Gibson Primer | CCTGCAGGTCGACTCTAGAGGATCCCTCCGCCCGACCTTTTTC |
| **26_hypo_up_rev** | Gibson Primer | ATGTGCGCACCCTGATTTCCGGAATACAAAATCTG |
| **26_hpyo_down_fwd** | Gibson Primer | GGAAATCAGGGTGCGCACATCAATACTCCG |
| **26_hypo_down_rev** | Gibson Primer | TTGTAAAACGACGGCCAGTGAATTCATCACCATCCGTGCGTGC |
| **26_hypo_Check_fwd** | Sequencing Primer | GGTGGACAACGGCGATGAAG |
| **26_hypo_Check_rev** | Sequencing Primer | AGTCGAGCGCACCACATCAG |
| **27_putA_up_fwd** | Gibson Primer | CCTGCAGGTCGACTCTAGAGGATCCGTTTGGAATTCACGGTTGGTTTCGTGGATCG |
| **27_putA_up_rev** | Gibson Primer | TGCCTTTCGACGCCCGCGCGGCCTCTAT |
| **27_putA_down_fwd** | Gibson Primer | CGCGCGGGCGTCGAAAGGCACGAAGTTCATCAACCTGGAC |
| **27_putA_down_rev** | Gibson Primer | TTGTAAAACGACGGCCAGTGAATTCGGCGCGCGCCTGATCGGA |
| **27_putA_Check_fwd** | Sequencing Primer | CTGACCAGGCTGTGGACAAG |
| **27_putA_Check_rev** | Sequencing Primer | GGCGCACGAGGTAAGAAATG |
| **28_Cu-ATPase_up_fwd** | Gibson Primer | CCTGCAGGTCGACTCTAGAGGATCCATTCGGGGCGGATTTTGTCCTCCAC |
| **28_Cu-ATPase_up_rev** | Gibson Primer | TCGCGATCTTCTCCGAGCGCGCCGCGAA |
| **28_Cu-ATPase_down_fwd** | Gibson Primer | GCGCTCGGAGAAGATCGCGATGACCAGCG |
| **28_Cu-ATPase_down_rev** | Gibson Primer | TTGTAAAACGACGGCCAGTGAATTCGGCCGAGAAGGTCGTCGAC |
| **28_Cu-ATPase Check fwd** | Sequencing Primer | GGCGAAGACCTCATCGATCC |
| **28_Cu-ATPase_Check_rev** | Sequencing Primer | ATCATGCTGTTGGGCCACTG |
| **29_helicase_up_fwd** | Gibson Primer | CCTGCAGGTCGACTCTAGAGGATCCCCAAGATGGGAGCCCACAATTC |
| **29_helicase_up_rev** | Gibson Primer | ACCGCTTTAATAATCCCGCTGCACAAGG |
| **29_helicase_down_fwd** | Gibson Primer | AGCGGGATTATTAAAGCGGTCGAGGTCTAAG |
| **29_helicase_down_rev** | Gibson Primer | TTGTAAAACGACGGCCAGTGAATTCCACGCTCCTCCAGACTTC |
| **29_helicase_Check_fwd** | Sequencing Primer | AACCAGGGCATGTTCCTTAG |
| **29_helicase_Check_rev** | Sequencing Primer | TGCCGATCCTACAAAGGTTC |
| **30_xylB_up_fwd** | Gibson Primer | CCTGCAGGTCGACTCTAGAGGATCCGAATGAGCTGTCGGCGGATATTTC |
| **30_xylB_up_rev** | Gibson Primer | CTAACAACCTCTCGGTAGCTTGATCCAGC |
| **30_xylB_down_fwd** | Gibson Primer | AGCTACCGAGAGGTTGTTAGAACGCGCGG |
| **30_xylB_down_rev** | Gibson Primer | TTGTAAAACGACGGCCAGTGAATTCATCTCCCGTGCCTGCAGC |
| **30_xylB_Check_fwd** | Sequencing Primer | AAGACAATGGCGGATCATCG |
| **30_xylB_Check_rev** | Sequencing Primer | GATGGATCGTGGACGCTATG |
| **del fasB_Check fwd** | Sequencing Primer | GGAGGATACATCCACGGTCATTG |
| **del fasB_Check rev** | Sequencing Primer | CGCTATGAGTTCAGGATGTTGATCG |
| **del2964_up_fwd** | Gibson Primer | CCTGCAGGTCGACTCTAGAGGATCCAGGACAATGGGGAAGAGAC |
| **del2964_up_rev** | Gibson Primer | GATTACTGATGCCGTTGAGACCATGAGAATTG |
| **del2964_down_fwd** | Gibson Primer | TCTCAACGGCATCAGTAATCGTGGACATGCATTCC |
| **del2964_down_rev** | Gibson Primer | TTGTAAAACGACGGCCAGTGAATTCAACCCAGCGATGGCCGTT |
| **del2964_Check_fwd** | Sequencing Primer | CCGCATCCCTGATATTTCGAACTG |
| **del2964_Check_rev** | Sequencing Primer | GTACACGTACTTGTACCACTGC |
| **del pks_up_fwd** | Gibson Primer | CCTGCAGGTCGACTCTAGAGGATCCACGCGCGATCTCTGAGTAC |
| **del pks_up_rev** | Gibson Primer | GAGCCAATCGAGTCGCCGCATTGATGAG |
| **del pks_down_fwd** | Gibson Primer | TGCGGCGACTCGATTGGCTCTGTTCCATATTG |
| **del pks_down_rev** | Gibson Primer | TTGTAAAACGACGGCCAGTGAATTCTGACATTGAAAAGCTCATCATC |
| **del pks_Check_fwd** | Sequencing Primer | CTGAGTACATCGCCAAGGAG |
| **del pks_Check_rev** | Sequencing Primer | TGGCACTGCACACGGTGTTG |
| **del emb_up_fwd** | Gibson Primer | CCTGCAGGTCGACTCTAGAGGATCCCATATGCTTATCCACGAGGTTG |
| **del emb_up_rev** | Gibson Primer | AGTCGGTGGTCTCTGGAATCCAGGGCATATG |
| **del emb_down_fed** | Gibson Primer | GATTCCAGAGACCACCGACTTGGCGCATAG |
| **del emb_down_rev** | Gibson Primer | TTGTAAAACGACGGCCAGTGAATTCACGCGCAAGATCTGCCGC |
| **del emb_Check_fwd** | Sequencing Primer | ATCTCCGACTGGTACTCATC |
| **del emb_Check_rev** | Sequencing Primer | CCGGAGCTGCACGTTATTAC |
| **del gltB_up_fwd** | Gibson Primer | CCTGCAGGTCGACTCTAGAGGATCCCTCATCCCAATTGGCGGTG |
| **del gltB_up_rev** | Gibson Primer | TTGCTGGGTCGAGTCCTTGTGGTTTCATGC |
| **del gltB_down_fed** | Gibson Primer | ACAAGGACTCGACCCAGCAATCAAGATCATGGAGGCAGTG |
| **del gltB_down_rev** | Gibson Primer | TTGTAAAACGACGGCCAGTGAATTCGCCAGCGGGGCCGGAACC |
| **del gltB_Check_fwd** | Sequencing Primer | AAGCTGCAACGCCTTCGATTTTTCC |
| **del gltB_Check_rev** | Sequencing Primer | GCCGTAGCGCATGAGGCCGCCGAGG |
| **del NCgl0552_up_fwd** | Gibson Primer | CCTGCAGGTCGACTCTAGAGGATCCAAAGTCATCAATTCCACG |
| **del NCgl0552_up_rev** | Gibson Primer | TCTGTGCAACCTCCACTGATGTTGTCATTTTC |
| **del NCgl0552_down_fwd** | Gibson Primer | ATCAGTGGAGGTTGCACAGATGGCACGC |
| **del NCgl0552_down_rev** | Gibson Primer | TTGTAAAACGACGGCCAGTGAATTCCTGCGTTGAAATCTCCGC |
| **del NCgl0552_Check_fwd** | Sequencing Primer | GTGTTTCTGCAAGCGGAATC |
| **del NCgl0552_Check_rev** | Sequencing Primer | GAAATCTCCGCCACCTTATC |
| **del NCgl1737_up_fwd** | Gibson Primer | CCTGCAGGTCGACTCTAGAGGATCCTGAACATTTGCTGCACGAATTTC |
| **del NCgl1737_up_rev** | Gibson Primer | GACAGTTTTAGGCGTAACCGAGAAGGGTG |
| **del NCgl1737_down_fwd** | Gibson Primer | CGGTTACGCCTAAAACTGTCGCAGTCAC |
| **del NCgl1737_down_rev** | Gibson Primer | TTGTAAAACGACGGCCAGTGAATTCAGGTAAGGGCTTTGATAAAG |
| **del NCgl1737_Check_fwd** | Sequencing Primer | TCGACGAACCTCGACTTCAG |
| **del NCgl1737_Check_rev** | Sequencing Primer | TGACGAGGTGCGTAGTGATG |
| **del NCgl2959_up_fwd** | Gibson Primer | CCTGCAGGTCGACTCTAGAGGATCCGCTGTGGCTGCGGCGATT |
| **del NCgl2959_up_rev** | Gibson Primer | AGTCCCAAATGAGCTTTGAGATGTTCATAATTTTTCCTAGATCCAATG |
| **del NCgl2959_down_fwd** | Gibson Primer | CTCAAAGCTCATTTGGGACTATGTGACCAAC |
| **del NCgl2959_down_rev** | Gibson Primer | TTGTAAAACGACGGCCAGTGAATTCCTGGAGTGGTGGGTGAAATC |
| **del NCgl2959_Check_fwd** | Sequencing Primer | TTTACGCTCCCACCAAATCC |
| **del NCgl2959_Check_rev** | Sequencing Primer | TGCTTCGGTGGTGCTGACTG |
| **del NRPS cps_Check fwd** | Sequencing Primer | GCAAACATAGTTCTAGATCAG |
| **del NRPS cps_Check rev** | Sequencing Primer | TGGATCGGGTGAGCACCTTTG |
| **del pknB_up_fwd** | Gibson Primer | CCTGCAGGTCGACTCTAGAGGATCCCTGGATCCGCCCGCACAA |
| **del pknB_up_rev** | Gibson Primer | CGTGATCGCTCTCTTCGAATTCGATCTCGCTGC |
| **del pknB_down_fwd** | Gibson Primer | ATTCGAAGAGAGCGATCACGAAGGTCAC |
| **del pknB_down_rev** | Gibson Primer | TTGTAAAACGACGGCCAGTGAATTCGCTAGCTGTTTCTGCTGTG |
| **del pknB_Check_fwd** | Sequencing Primer | AACAACTGCCGCAGCACAAC |
| **del pknB_Check_rev** | Sequencing Primer | GAAACGCGGTTCCGGCATTG |
| **del putA_up_fwd** | Gibson Primer | CCTGCAGGTCGACTCTAGAGGATCCTGGCCACTGGTGGTGACATC |
| **del putA_up_rev** | Gibson Primer | CGTGGAAGGCCAGATTCATCGACGTCATGGTG |
| **del putA_down_fwd** | Gibson Primer | GATGAATCTGGCCTTCCACGAGTTGGCG |
| **del putA_down_rev** | Gibson Primer | TTGTAAAACGACGGCCAGTGAATTCTTACTGCCCCGAGCATAGG |
| **del putA_Check_fwd** | Sequencing Primer | ATGTCGAATACCGGATCGTC |
| **del putA_Check_rev** | Sequencing Primer | GTCACGCCGTGCTCCATTTC |
| **del ggtB_up_fwd** | Gibson Primer | CCTGCAGGTCGACTCTAGAGGATCCACTGCTTCCGAGGATCTG |
| **del ggtB_up_rev** | Gibson Primer | GACCCAAAGCGATCCACGTAGAGAAGGC |
| **del ggtB_down_fwd** | Gibson Primer | TACGTGGATCGCTTTGGGTCACTTTCATC |
| **del ggtB_down_rev** | Gibson Primer | TTGTAAAACGACGGCCAGTGAATTCCTGCGAATAAATAGGAATAGTTAAAAAC |
| **del ggtB_Check_fwd** | Sequencing Primer | GATGGTCAGCCAAGAAATCC |
| **del ggtB_Check_rev** | Sequencing Primer | TTGCGGAATCGGTTGATTCG |
| **del NCGL2981_Check2_fwd** | Sequencing Primer | CAGCAGTCCCGTTTGGTTAC |
| **del NCGL2981_Check2_rev** | Sequencing Primer | ACCGCACCCAACACGATTTC |
| **delCGP3_D20_Check fwd** | Sequencing Primer | CTACCGTTACAGCAGCTCAG |
| **delCGP3_D20_Check rev** | Sequencing Primer | CCGCACTGGAATTAGCTTTG |
| **del pyk1_up_fwd** | Gibson Primer | CGACGCGAAGGCTAACTGCATCCATG |
| **del pyk1_up_rev** | Gibson Primer | AGACGGCATGGATTCACGTCCACCTTCTTG |
| **del pyk1_down_fwd** | Gibson Primer | ACGTGAATCCATGCCGTCTTCTACCAAAC |
| **del pyk1_down_rev** | Gibson Primer | GGCATCCGAATCAACACCATC |
| **del pyk1_Check fwd** | Sequencing Primer | CCATTGGTTCAACGCTAAGG |
| **del pyk1_Check rev** | Sequencing Primer | AGGGCATTGATGGAGAAACG |
| **del pyk2 up fwd** | Gibson Primer | CAGGTCGACTCTAGAGGATCCTTAGCCGAATGCTGGACAAAG |
| **del pyk2 up rev** | Gibson Primer | GGGTAGGTGATTTGAATTTGTCTGGTCAAACTCATTCATTTGAGCTCC |
| **del pyk2 down fwd** | Gibson Primer | ACAAATTCAAATCACCTACCCCTGCTGCTGCGCAAGGTGAAG |
| **del pyk2 down rev** | Gibson Primer | AAAACGACGGCCAGTGAATTCAATCCTTGAAGATCCAAATGTTG |
| **del pyk2 Check fwd** | Sequencing Primer | CAGACAGGGAGGACAAGAATC |
| **del pyk2 Check rev** | Sequencing Primer | ACACGGCGACACTGAACTTCC |


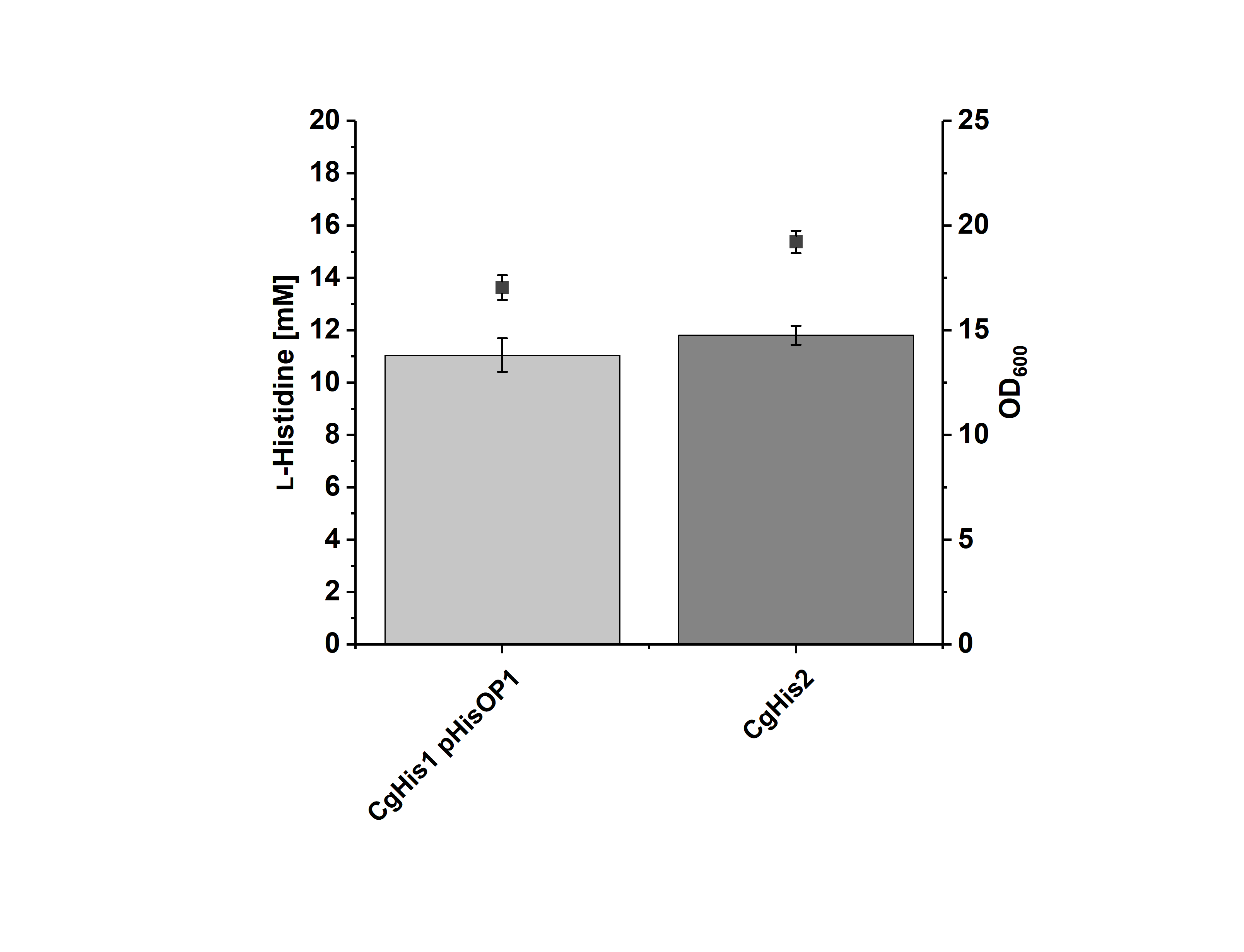


Figure S1: Comparison of *C. glutamicum* CgHis1 pHisOP1 and its variant *C. glutamicum* CgHis2, which served as screening host. Results from microtiter cultivations regarding l-histidine titer (bars) and biomass formation (dots, OD_600_). Error bars represent biological triplicates.


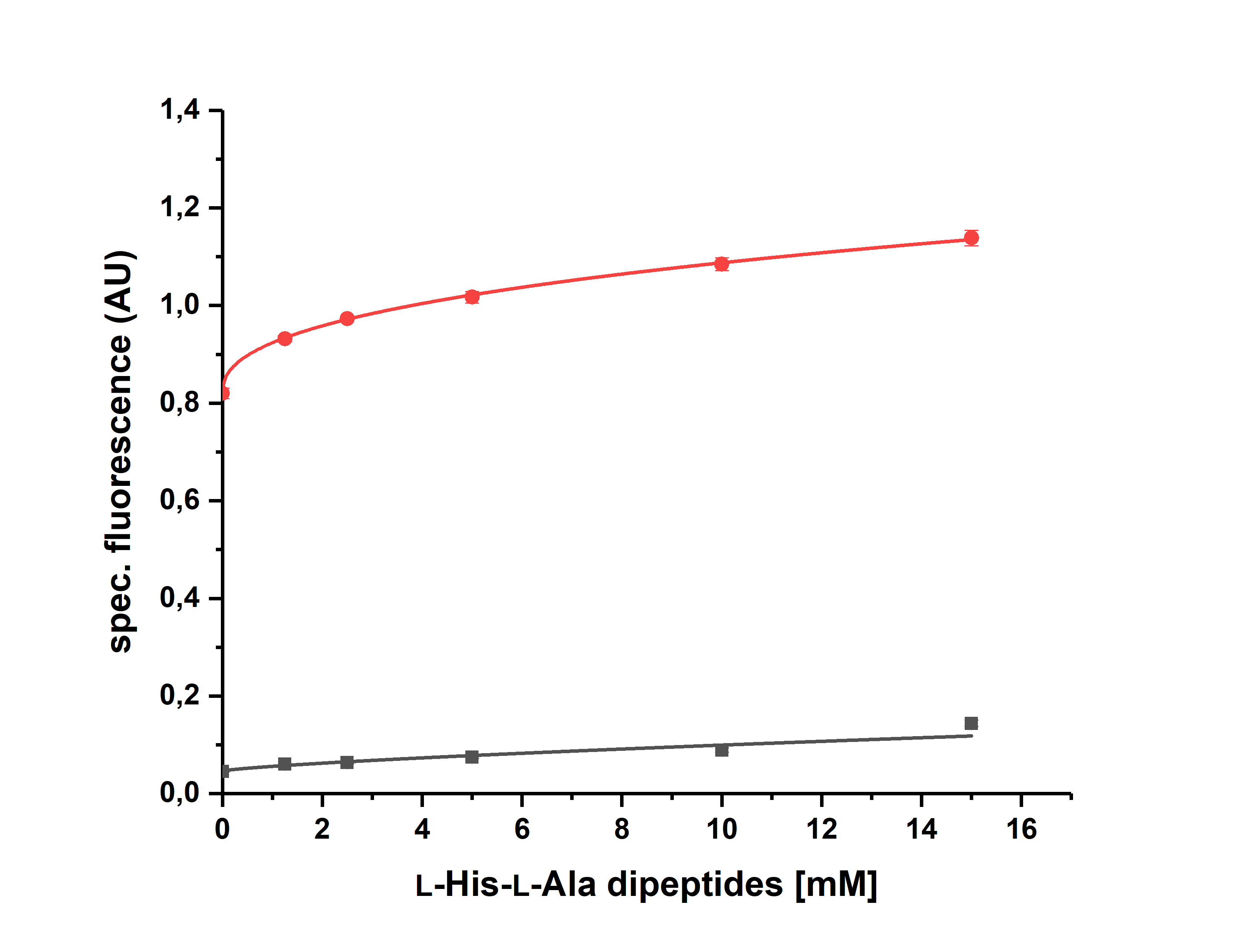


Figure S2: Specific fluorescence response of biosensor-harboring *C. glutamicum* strains upon initial dipeptide supplementation. An enhanced l-histidine productivity of *C. glutamicum* CgHis2 (red) and pSenHis[*hisEG*]-harboring *C. glutamicum* wild type cells (grey) was simulated supplementing l-histidine at concentrations of 0, 1.25, 2.5, 5, 10 and 15 mM l-His-l-Ala dipeptides, respectively. l‑Ala-l-Ala dipeptides were always added to final dipeptide concentration of 15 mM to always supplement the same amount of dipeptides. Standard deviations represent three independent biological replicates. All microtiter plate cultivations were performed with following parameters: 30 °C, 900 rpm and 85 % rel. humidity; 800 µl culture volume (defined CGXII medium, 4 % glucose and ± 15 µg mL^-1^ kanamycin) in Flowerplates and 48 h cultivation time.


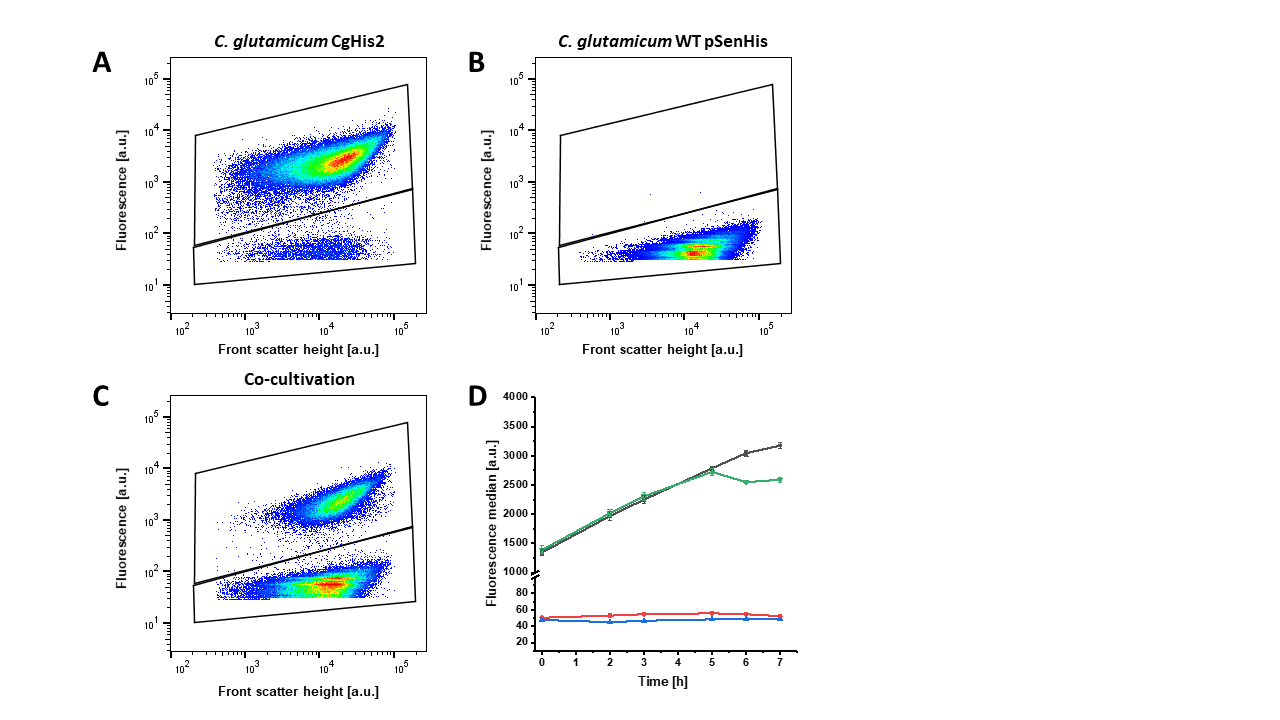


**Fig. S3:** **Biosensor crosstalk – Co-cultivation of *C. glutamicum* CgHis2 and *C. glutamicum* wild type pSenHis**. Scatter plots for **A)** *C. glutamicum* CgHis2, **B)** *C. glutamicum* wildtype pSenHis as reporter strain and **C)** the co-cultivation of both strains were derived from the FACS analysis of 100,000 events, respectively. The upper and lower gates were used to monitor the populations’ fluorescence median over a cultivation time of seven hours. **D)** Progression of fluorescence medians of *C. glutamicum* CgHis2 control (green, from upper gate in A); *C. glutamicum* WT pSenHis control (blue, from lower gate in B); *C. glutamicum* CgHis2 subpopulation of co-cultivation (black, from upper gate in C); *C. glutamicum* WT pSenHis subpopulation of co-cultivation (red, from lower gate in C). Data represent average values and standard deviation of three independent technical replicates.

## Screening workflow for the identification of improved l-histidine producing variants


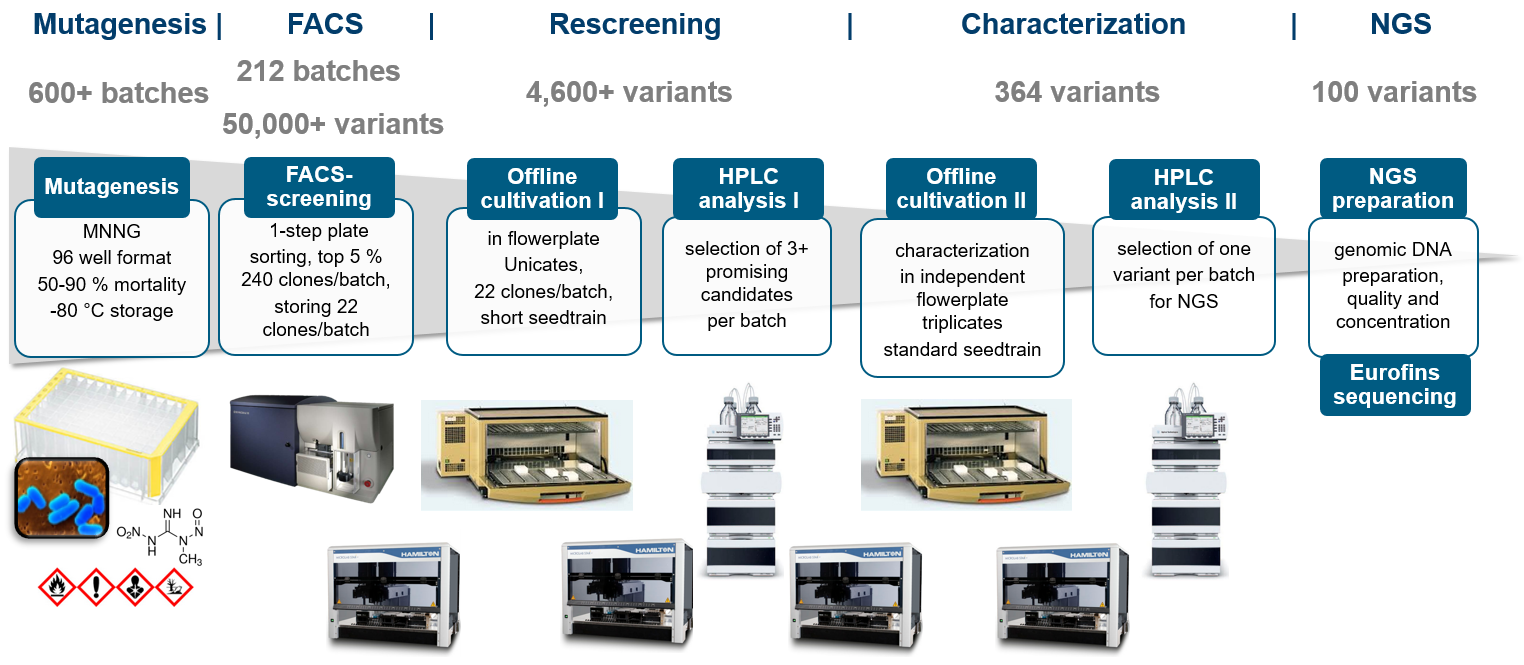


**Figure S4: Schematic overview of the screening workflow for the identification of significantly improved L-histidine producing *C. glutamicum* CgHis2 strain variants**. More than 600 *C. glutamicum* CgHis2 cultures were treated with MNNG in a multiplexed random genome mutagenesis approach. Subsequently, 212 mutagenized cultures were screened by FACS for single cells with increased fluorescence, thereby isolating more than 50,000 *C. glutamicum* CgHis2 strain variants. Rescreenings comprising an individual cultivation of more than 4,600 FACS-isolated *C. glutamicum* CgHis2 variants were performed. Of these, 364 identified strain variants with a significantly improved production phenotype were subjected to more detailed characterizations to verify the increased l-histidine accumulation. For high accuracy, all cultivation and HPLC analysis steps were performed using robotics. Finally, significantly improved L-histidine production was verified for 100 *C. glutamicum* CgHis2 strain variants. Their genomes were sequenced and comparative and combinatorial genome analyses were performed.

## Genome sequencing and automated analysis of 100 genome sequences


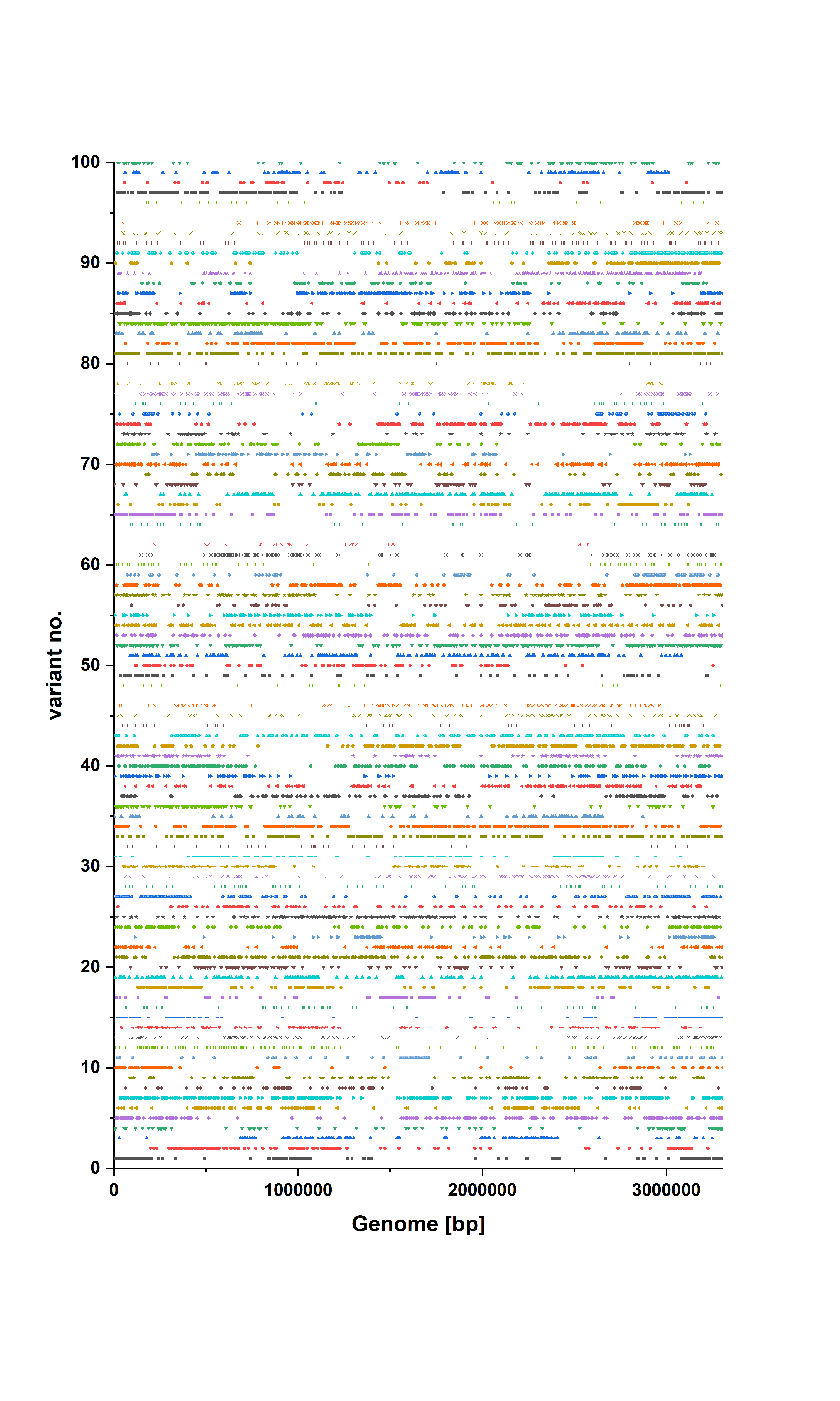


**Figure S5:** Individual SNP-distribution across the genomes of all 100 FACS-isolated and L-histidine producing *C. glutamicum* CgHis2 variants. Respective strain variants are listed 0-100 according to Table S1.

**Figure S6:** **Mutational bias of MNNG-mutagenesis.** MNNG predominantly introduces GC→AT transitions, which affects the spectrum of observed amino acid substitutions in the 100 CgHis2 genomes dataset. Amino acid substitutions are displayed as one letter code (i.e. AV, Alanine→Valine).

**Table S3:** **Identified mutations in already known genetic targets contributing to l-histidine production from 100 improved *C. glutamicum* CgHis2 variants**. The genes *hisG*, from which a multitude of variants was already described [5,6], as well as *hisD*, *hisN*, *hisC*, *hisA*, *hisH*, *hisF*, *hisE* are part of the l-histidine biosynthesis pathway. *purA*, *purB* and *purH* are part of the de-novo biosynthesis pathway of purines [7–10]. *glyA* is essential for providing C_1_-compunds (mTHF, fTHF) needed for conversion of l‑histidine pathway intermediate AICAR towards purine and hence ATP biosynthesis as l‑histidine precursor [7,9,11]. *hisG**, *hisG*(S143F/ΔC).

| **#** | **Locus tag** | ***Gene*** | **Enzyme** | **Nonsynonymous mutations**  **in x variants** |
| --- | --- | --- | --- | --- |
| 1 | NCgl1447 | *hisG** | ATP-phosphoribosyl-transferase | 9 |
| 2 | NCgl2021 | *hisD* | Histidinol dehydrogenase | 4 |
| 3 | NCgl0765 | *hisN* | Histidinol-phosphatase | 4 |
| 4 | NCgl2020 | *hisC* | histidinol-phosphate aminotransferase | 3 |
| 5 | NCgl2015 | *hisA* | Phosphoribosylformimino-5-aminoimidazole carboxamide ribotide isomerase | 3 |
| 6 | NCgl2016 | *hisH* | imidazole glycerol phosphate synthase subunit | 1 |
| 7 | NCgl2013 | *hisF* | imidazole glycerol phosphate synthase subunit | 1 |
| 8 | NCgl1448 | *hisE* | phosphoribosyl-ATP pyrophosphatase | 1 |
| 9 | NCgl2669 | *purA* | adenylosuccinate synthetase | 2 |
| 10 | NCgl2509 | *purB* | adenylosuccinate lyase | 5 |
| 11 | NCgl0827 | *purH* | bifunctional phosphoribosylaminoimidazolecarboxamide formyltransferase/IMP cyclohydrolase | 5 |
| 12 | NCgl0954 | *glyA* | serine hydroxymethyltransferase | 3 |

## Identification of beneficial mutations and reverse engineering

**Table S4: Hotspot genes in *C. glutamicum* CgHis2 identified by computational analysis of the FAAMS dataset.** Depicted are the genomic annotation and locus tags of the respective genes as well as the number of nonsynonymous SNPs (numSNP) of all 100 *C. glutamicum* CgHis2 strain variants in the particular gene and number of strain variants harboring nonsynonymous SNPs in the particular gene (numCLO).

| **#** | **Annotation** | | **Locus tag** | | **Gene** | **numSNP** | **numCLO** | |
| --- | --- | --- | --- | --- | --- | --- | --- | --- |
| **1** | CGL_RS12390 | NCgl2409 | | *fasB* | | 37 | 24 |  |
| **2** | CGL_RS15265 | NCgl2964 | |  | | 28 | 23 |  |
| **3** | CGL_RS04205 | NCgl0802 | | *fasA* | | 26 | 22 |  |
| **4** | CGL_RS14305 | NCgl2773 | | *pks* | | 27 | 21 |  |
| **5** | CGL_RS00975 | NCgl0184 | | *emb* | | 27 | 20 |  |
| **6** | *gltB* | NCgl0181 | | *gltB* | | 22 | 19 |  |
| **7** | CGL_RS02900 | NCgl0552 | |  | | 21 | 19 |  |
| **8** | CGL_RS13520 | NCgl2618 | | *cps* | | 23 | 18 |  |
| **9** | *pknB* | NCgl0040 | | *pknB* | | 20 | 17 |  |
| **10** | CGL_RS15240 | NCgl2959 | |  | | 19 | 17 |  |
| **11** | CGL_RS09035 | NCgl1737 | |  | | 18 | 17 |  |
| **12** | CGL_RS12520 | NCgl2433 | | *dinG* | | 16 | 15 |  |
| **13** | CGL_RS15355 | NCgl2981 | |  | | 16 | 15 |  |
| **14** | *ggt* | NCgl0916 | | *ggtB* | | 15 | 14 |  |
| **15** | CGL_RS03670 | NCgl0705 | |  | | 18 | 13 |  |
| **16** | CGL_RS11935 | NCgl2324 | | *benR* | | 15 | 13 |  |
| **17** | CGL_RS00545 | NCgl0098 | | *putA* | | 14 | 13 |  |
| **18** | CGL_RS00870 | NCgl0163 | |  | | 14 | 13 |  |
| **19** | CGL_RS12940 | NCgl2503 | | *nuc* | | 14 | 13 |  |
| **20** | CGL_RS13350 | NCgl2585 | | *clpC* | | 14 | 13 |  |
| **21** | CGL_RS14755 | NCgl2859 | |  | | 17 | 12 |  |
| **22** | CGL_RS02505 | NCgl0472 | | *rpoC* | | 16 | 12 |  |
| **23** | CGL_RS13580 | NCgl2628 | |  | | 15 | 12 |  |
| **24** | CGL_RS13605 | NCgl2633 | | *mrpA* | | 14 | 12 |  |
| **25** | CGL_RS05640 | NCgl1085 | |  | | 13 | 12 |  |
| **26** | *iolD* | NCgl0159 | | *iolD* | | 13 | 12 |  |
| **27** | CGL_RS08860 | NCgl1702 | |  | | 14 | 11 |  |
| **28** | CGL_RS02075 | NCgl0394 | |  | | 13 | 11 |  |
| **29** | CGL_RS04840 | NCgl0927 | |  | | 13 | 11 |  |
| **30** | CGL_RS05800 | NCgl1117 | |  | | 13 | 11 |  |
| **31** | CGL_RS13165 | NCgl2548a | |  | | 13 | 11 |  |
| **32** | CGL_RS03135 | NCgl0599 | |  | | 12 | 11 |  |
| **33** | CGL_RS03195 | NCgl0611 | | *dnaE2* | | 12 | 11 |  |
| **34** | CGL_RS04255 | NCgl0812 | |  | | 12 | 11 |  |
| **35** | CGL_RS07785 | NCgl1494a | |  | | 12 | 11 |  |
| **36** | CGL_RS13380 | NCgl2591 | |  | | 12 | 11 |  |
| **37** | CGL_RS14975 | NCgl2903 | |  | | 12 | 11 |  |
| **38** | *glpK* | NCgl2790 | | *glpK* | | 12 | 11 |  |
| **39** | *prpB* | NCgl0629 | | *prpB* | | 12 | 11 |  |
| **40** | CGL_RS00515 | NCgl0092 | |  | | 11 | 11 |  |
| **41** | CGL_RS10665 | NCgl2068 | | *ileS* | | 11 | 11 |  |
| **42** | CGL_RS14485 | NCgl2809 | | *pyk2* | | 11 | 11 |  |
| **43** | *pepN* | NCgl2340 | | *pepN* | | 11 | 11 |  |
| **44** | CGL_RS14385 | NCgl2789 | | *psp5* | | 18 | 10 |  |
| **45** | CGL_RS02500 | NCgl0471 | | *rpoB* | | 15 | 10 |  |
| **46** | CGL_RS07320 | NCgl1407 | | *thiD1* | | 15 | 10 |  |
| **47** | CGL_RS04190 | NCgl0799 | | *mctC* | | 13 | 10 |  |
| **48** | CGL_RS03440 | NCgl0659 | | *pyc* | | 13 | 10 |  |
| **49** | CGL_RS03060 | NCgl0584 | |  | | 12 | 10 |  |
| **50** | *mfd* | NCgl0924 | | *mfd* | | 12 | 10 |  |
| **51** | CGL_RS02370 | NCgl0450 | | *menD* | | 11 | 10 |  |
| **52** | CGL_RS05435 | NCgl1044 | |  | | 11 | 10 |  |
| **53** | CGL_RS08810 | NCgl1692 | |  | | 11 | 10 |  |
| **54** | CGL_RS11220 | NCgl2185 | | *phoD* | | 11 | 10 |  |
| **55** | CGL_RS12955 | NCgl2507 | | *ptrB* | | 11 | 10 |  |
| **56** | CGL_RS14895 | NCgl2887 | |  | | 11 | 10 |  |
| **57** | CGL_RS14915 | NCgl2891 | |  | | 11 | 10 |  |
| **58** | *gabT* | NCgl0462 | | *gabT* | | 11 | 10 |  |
| **59** | *hrpB* | NCgl0139 | | *hrpB* | | 11 | 10 |  |
| **60** | *topA* | NCgl0304 | | *topA* | | 11 | 10 |  |
| **61** | CGL_RS00340 | NCgl0060 | |  | | 10 | 10 |  |
| **62** | CGL_RS01805 | NCgl0340 | | *capD* | | 10 | 10 |  |
| **63** | CGL_RS03515 | NCgl0674 | | *wbpC* | | 10 | 10 |  |
| **64** | CGL_RS09170 | NCgl1767 | |  | | 10 | 10 |  |
| **65** | CGL_RS12690 | NCgl2464 | |  | | 10 | 10 |  |
| **66** | CGL_RS14285 | NCgl2769 | | *mmpL1* | | 10 | 10 |  |
| **67** | CGL_RS14880 | NCgl2884 | | *mrcB* | | 10 | 10 |  |
| **68** | CGL_RS14925 | NCgl2893 | |  | | 10 | 10 |  |
| **69** | *fusA* | NCgl0478 | | *fusA* | | 10 | 10 |  |
| **70** | CGL_RS15125 | NCgl2933 | | *ulaA* | | 10 | 10 |  |
| **71** | *xylB* | NCgl0111 | | *xylB* | | 10 | 10 |  |

**Data S1: Computational analysis of FAAMS data.**

**Table S5:** **List of SNPs identified in 100 independently isolated l‑histidine producing *C. glutamicum* CgHis2 variants, selected for individual reconstruction in the *C. glutamicum* CgHis2 starting strain.**

| **#** | **Locus tag** | ***Gene*** | **Enzyme** | **Hotspot signifi-cance** | **Increase of l-His-prod. in the resp. FACS-isolated variant [%]** | **Amino**  **acid**  **substitution** |
| --- | --- | --- | --- | --- | --- | --- |
| **1** | NCgl0098 | *putA* | proline dehydrogenase/delta-1-pyrroline-5-carboxylate dehydrogenase | 13 | 15% | P217S |
| **2** | NCgl0111 | *xylB* | Xylulose kinase | 10 | 16% | G55R |
| **3** | NCgl0159 | *iolD* | putative acetolactate synthase protein | 12 | 31% | S481F |
| **4** | NCgl0181 | *gltB* | alpha subunit of glutamate synthase | 19 | 68% | P988S |
| **5** | NCgl0181 | *gltB* | alpha subunit of glutamate synthase | 19 | 19% | G1106D |
| **6** | NCgl0184 | *emb* | arabinosyl transferase C | 20 | 32% | T529I  T539I |
| **7** | NCgl0184 | *emb* | arabinosyl transferase C | 20 | 47% | G477E |
| **8** | NCgl0552 |  | type VII secretion protein eccC / DNS segregation ATPase | 19 | 11% | P823S |
| **9** | NCgl0552 |  | type VII secretion protein eccC / DNS segregation ATPase | 19 | 29% | G432D |
| **10** | NCgl0659 | *pyc* | pyruvate carboxylase | 10 | 35% | A764V |
| **11** | NCgl0705 |  | Probable ATP-dependent helicase | 13 | 34% | S1847N |
| **12** | NCgl0802 | *fasA* | fatty acid synthase A | 22 | 38% | A2702T |
| **13** | NCgl0802 | *fasA* | fatty acid synthase A | 22 | 36% | P783S |
| **14** | NCgl2409 | *fasB* | fatty acid synthase B | 24 | 36% | G2762D |
| **15** | NCgl2409 | *fasB* | fatty acid synthase B | 24 | 24% | G1921E |
| **16** | NCgl2618 | *cps* | non-ribosomal peptide synthase | 18 | 34% | G987D |
| **17** | NCgl2633 | *mrpA* | NADH ubiquinone oxidoreductase subunit 5 (chain L)/multisubunit Na+/H+ antiporter, A subunit | 12 | 45% | L42F |
| **18** | NCgl2773 | *pks* | type 1 polyketide synthase | 21 | 18% | A1525V |
| **19** | NCgl2773 | *pks* | type 1 polyketide synthase | 21 | 31% | D1186N |
| **20** | NCgl2789 |  | hypothetical protein | 10 | 18% | S265N |
| **21** | NCgl2809 | *pyk2* | pyruvate kinase 2 | 11 | 21% | T357I |
| **22** | NCgl2859 |  | probable cation-transporting ATPase transmembrane protein | 12 | 16% | S372F |
| **23** | NCgl2933 | *ulaA* | ascorbate-specific PTS system enzyme IIC | 10 | 28% | V219I |
| **24** | NCgl2959 |  | phosphoesterase | 17 | 43% | D1453N |
| **25** | NCgl2959 |  | phosphoesterase | 17 | 43% | G870D |
| **26** | NCgl2964 |  | DEAD/DEAH box helicase | 23 | 21% | E512K |
| **27** | NCgl2964 |  | DEAD/DEAH box helicase | 23 | 26% | P863S |
| **28** | NCgl2981 |  | hypothetical protein | 15 | 45% | D735G |

**Data S2: Rationale for selection of three SNPs for reconstruction in *C. glutamicum* CgHis2.**

**NCgl2981-D735G:** This mutation originates from *C. glutamicum* CgHis 2 11-10-7-14, which was characterized by 45% His-increase and 14% biomass increase, low abundance of additional mutations in other assumed hotspot genes, a potential high effect on the protein structure due to the D735G substitution, no information on protein structure or function and hence making it the best guess of the identified mutations in this gene.

**Pks-D1186N:** This mutation originates from *C. glutamicum* CgHis 2 7-40-10-2, which was characterized by an 31% His-increase and 13% biomass increase, medium abundance of mutations in other hotspot genes but nonsense codon in *fasB,* hence a potential cooperative mutation in cell wall synthesis. Pks is an interesting target for a non-essential carbon sink under production conditions.

**Cps-G987D:** This mutation chosen from *C. glutamicum* CgHis 2 7-50-8-15, which was characterized by an 34% His-increase and only 2% biomass decrease, low abundance of mutations in other hotspot genes of this variant, potential high effect of the G987D substitution on the unknown structure, but no information on protein structure/function making it the best guess of the identified mutations in this gene.


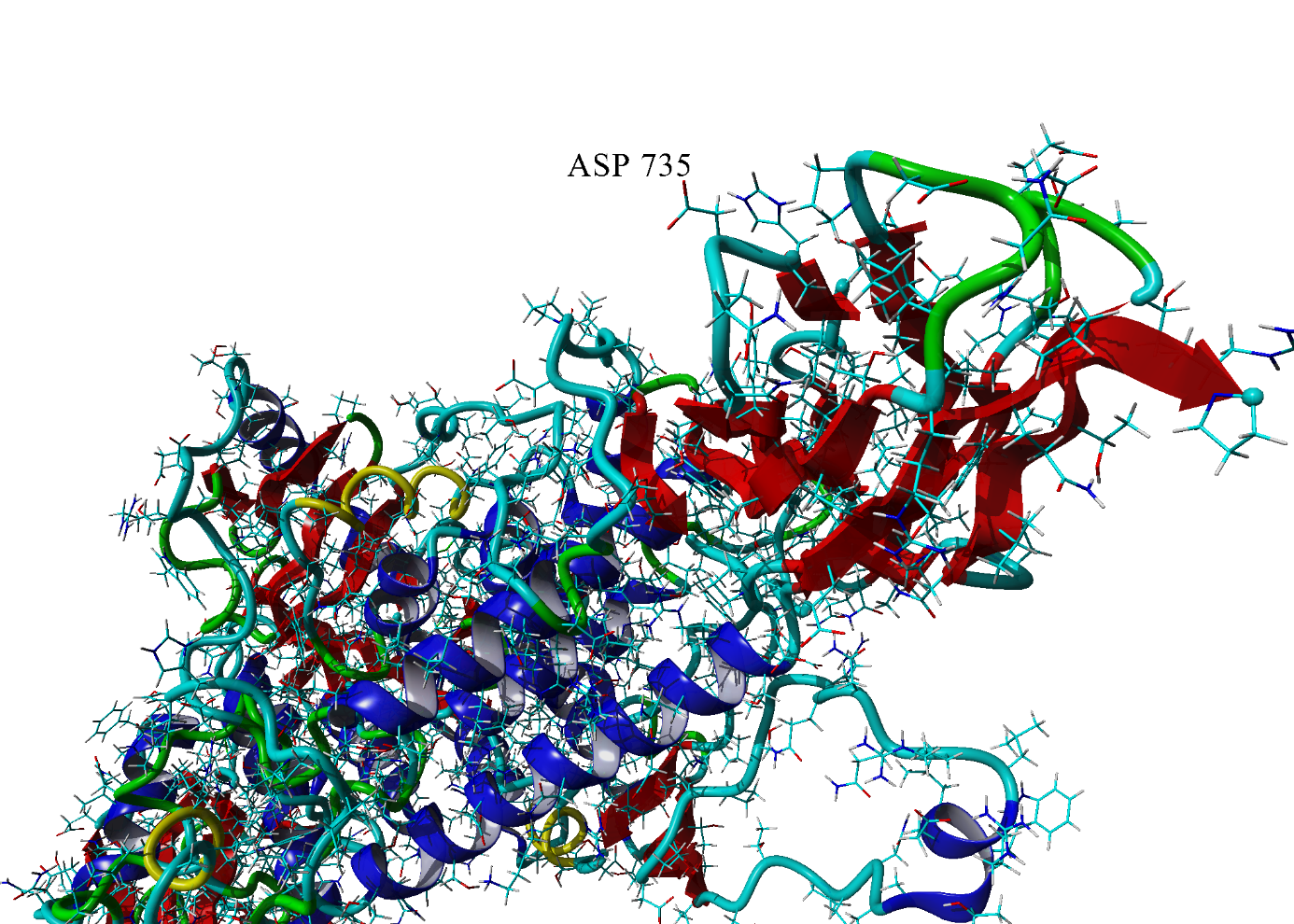


Figure S7: Predicted structure of NCgl2981 and position of the D735 residue as calculated by AlphaFold [12].

## Combination of beneficial mutations boosts l-histidine production


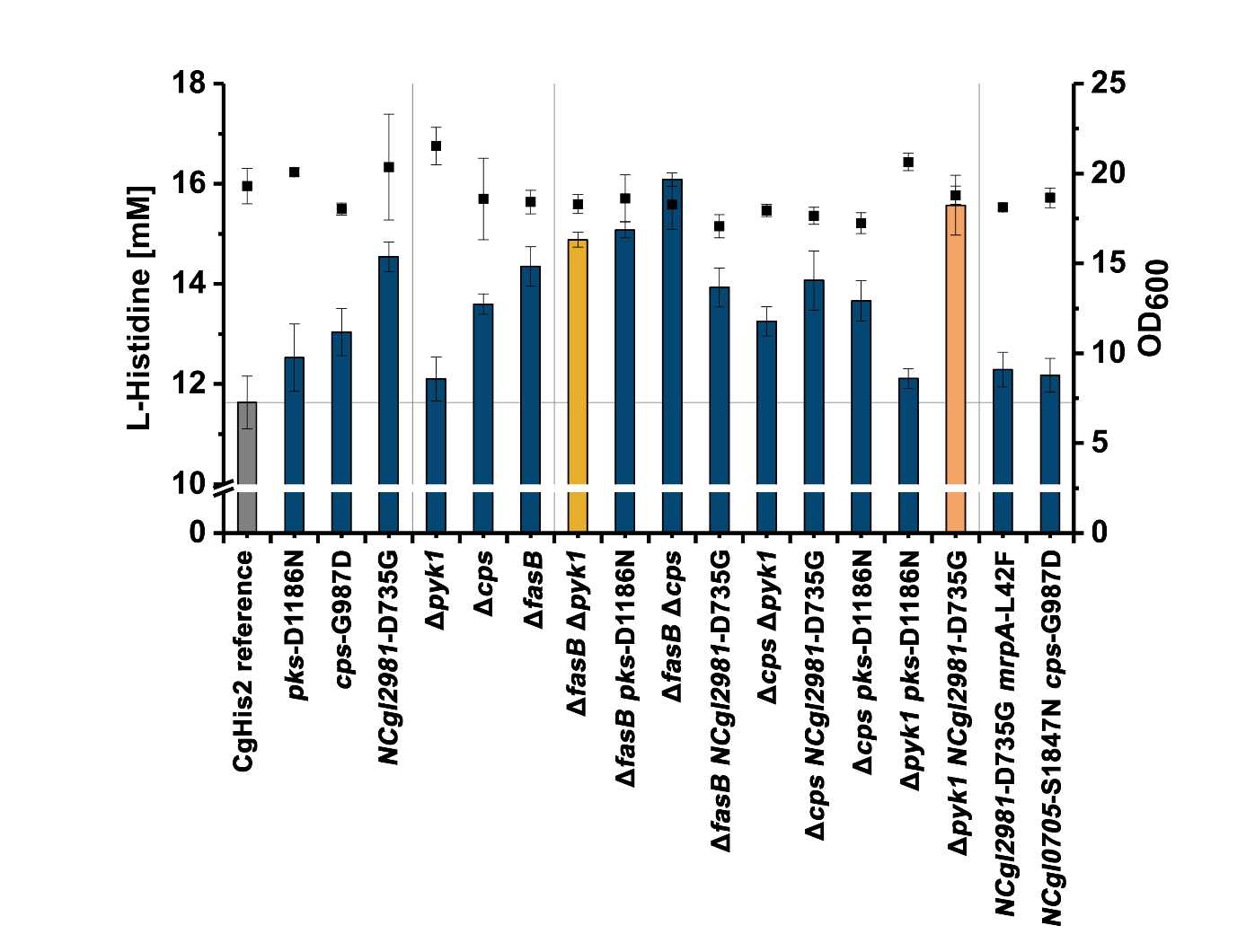


Figure S8: l-histidine production performance of reverse engineered CgHis2 single mutant- and double mutant strains. Whereas the *C. glutamicum* CgHis2 *ΔfasB* *Δcps* lineage could not be further improved by additive genomic modifications, did the *C. glutamicum* CgHis2 *ΔfasB Δpyk1* lineage (yellow) show further improvements and yielded the best-performing quadruple strain variant *C. glutamicum* CgHis2 *ΔfasB Δpyk1 NCgl2981*-D735G *pks-*D1186N (Figure 4). Interestingly, also the *C. glutamicum* CgHis2 *Δpyk1 NCgl2981-D735G* lineage (orange) outperformed the single mutant strains and could be further improved to the second best-performing strain *C. glutamicum* CgHis2 *Δcps Δpyk1 NCgl2981*-D735G (Suppl. Fig. 6). Data represent average values and standard deviation of three independent technical replicates.


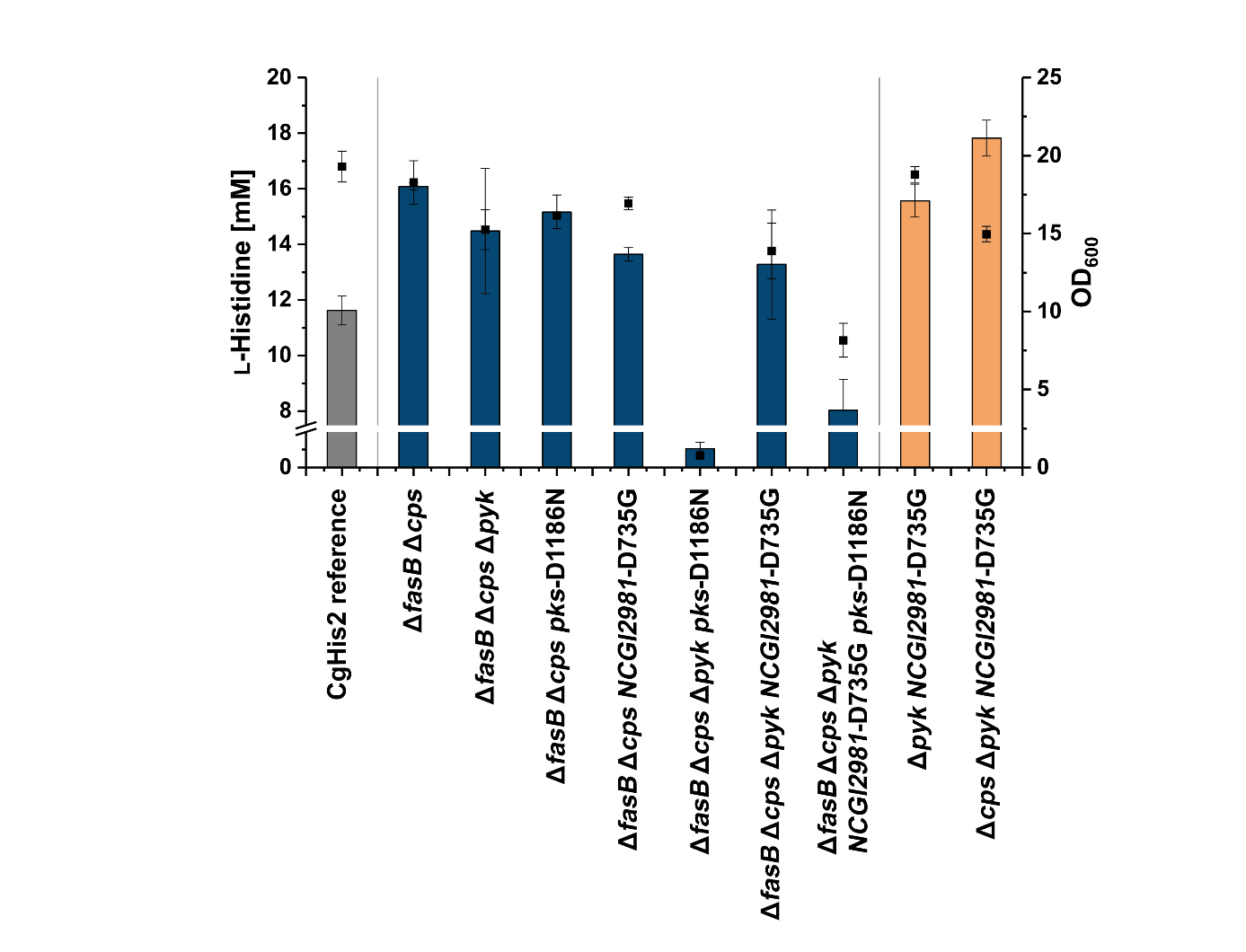


**Figure S9:** **l-histidine production performance of reverse engineered CgHis2 variants in comparison to the CgHis2 reference strain.** *C. glutamicum* CgHis2 Δ*fasB* Δ*cps*-derived strains (blue) and strains of the alternative lineage starting from *C. glutamicum* CgHis2 Δ*pyk1* *NCgl2981*-D735G (orange) are depicted. *Δpyk* always refers to *pyk1.* Data represent average values and standard deviation of three independent technical replicates.

**A**


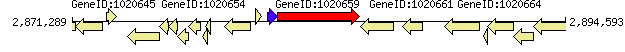


**B**


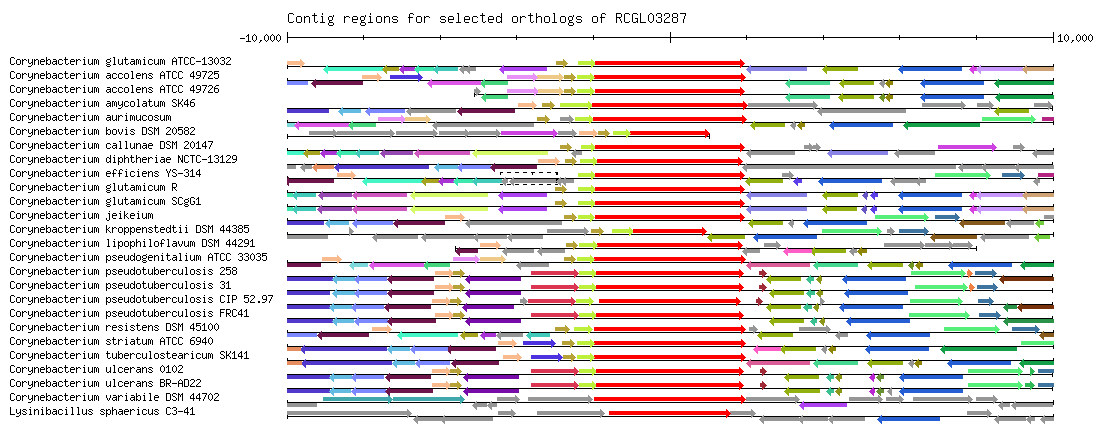


**Figure S10:** **A)** Genomic position of the *cps*-gene encoding the non-ribosomal peptide synthase (red) with an unknown MarR-type regulator gene located directly upstream (blue) in *C. glutamicum* ATCC 13032. **B)** The position of the *cps*-gene and the gene of the MarR-type regulator gene are highly conserved among various *Corynebacterium* species. Graphics generated by the ERGO systems biology informatics toolkit (Igenbio Inc., Chicago, IL, USA).

**A**


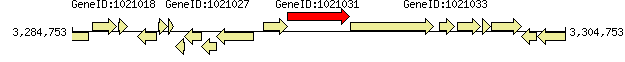


**B**


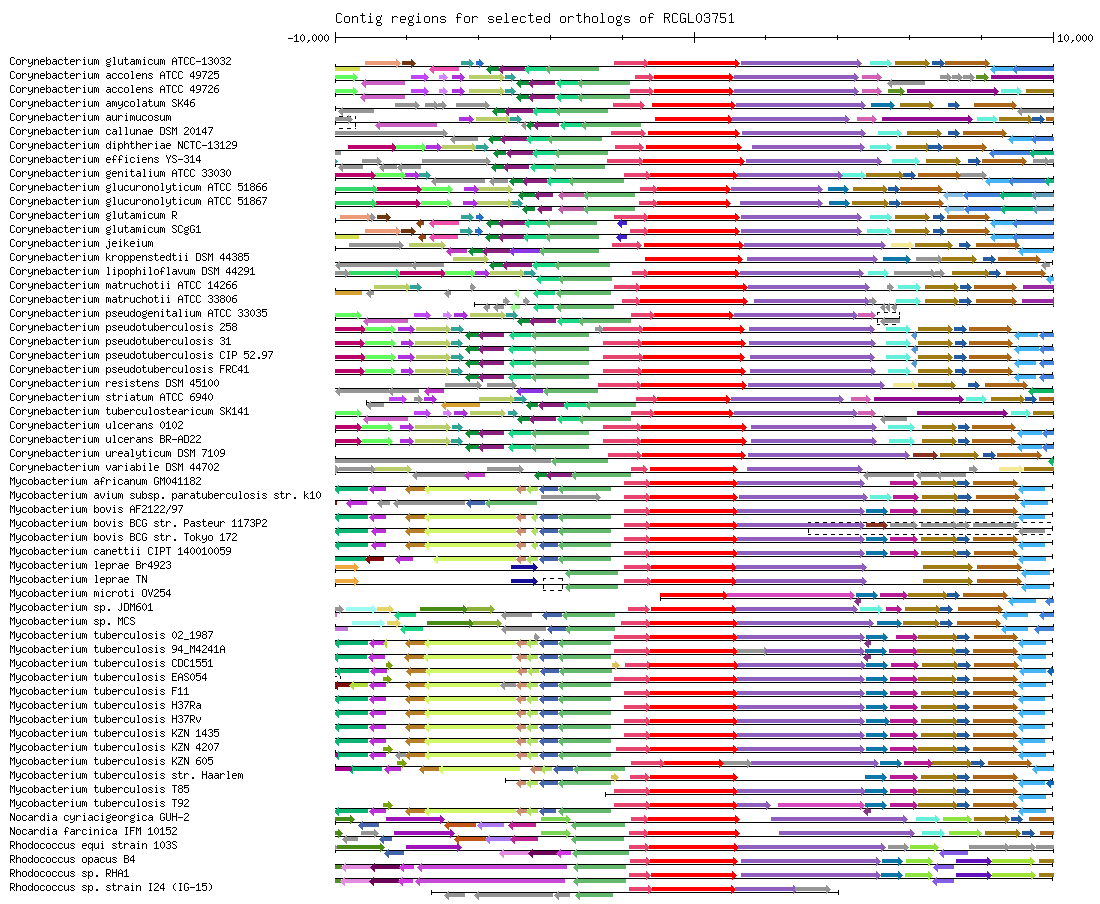


**Figure S11: A)** Genomic position of the *NCgl2981* gene (red) of unknown function in *C. glutamicum* ATCC 13032. **B)** *NCgl2981* (red) is highly conserved among various *Corynebacterium* and *Mycobacterium* species. Graphics generated by the ERGO systems biology informatics toolkit (Igenbio Inc., Chicago, IL, USA).
